# Correlation of brain injury biomarkers with brain dysfunction, brain injury, and outcomes in critically ill patients: a post hoc exploratory analysis

**DOI:** 10.1007/s15010-026-02790-2

**Published:** 2026-04-13

**Authors:** M. Rühlmann, L. Xu, M. Bauer, T. Lehmann, G. Panagiotou, S. Neugebauer, M. Kiehntopf, F. Klawitter, J. Ehler

**Affiliations:** 1 Department of Anesthesiology and Intensive Care Medicine, University Hospital Jena, Am Klinikum 1, 07747 Jena, Germany; 2 Department of Microbiome Dynamics, Leibniz Institute for Natural Product Research and Infection Biology - Hans Knöll Institute (Leibniz-HKI), Jena, Germany; 3 Institute of Clinical Chemistry and Laboratory Diagnostics, Am Klinikum 1, 07747 Jena, Germany; 4 Department of Anesthesiology, Intensive Care Medicine and Pain Therapy, University Medical Center Rostock, Schillingallee 35, 18057 Rostock, Germany; 5 Cluster of Excellence Balance of the Microverse, Friedrich Schiller University, Jena, Germany; 6 Department of Medicine, The University of Hong Kong, Hong Kong Special Administrative Region, China; 7 Faculty of Biological Sciences, Friedrich Schiller University, Jena, Germany; 8 Institute of Medical Statistics, Computer Sciences and Data Science, Bachstraße 18, 07743 Jena, Germany

**Keywords:** Delirium, Critical illness, Biomarkers, Brain injuries, Intensive care medicine, Sepsis, Neurocognitive disorders

## Abstract

**Purpose:**

Clinical assessment of brain dysfunction in critically ill patients is frequently limited by impaired consciousness and poor compliance. Blood-based biomarkers may facilitate detection of neurocognitive impairment, quantify structural brain injury, and improve prognostication. This study evaluated the potential diagnostic role of validated brain injury biomarkers compared with routine diagnostics in critically ill patients.

**Methods:**

We performed a single-center post hoc analysis of a prospective observational sepsis study conducted in two perioperative ICUs. Critically ill patients with and without sepsis were included. Delirium was assessed using validated tools and structural brain injury was evaluated from radiology reports. Biomarkers—neurofilament light chain (NfL), ubiquitin carboxy-terminal hydrolase L1 (UCH-L1), glial fibrillary acidic protein (GFAP) and Tau—were measured at two time points (enrollment and day 7). Neurological outcome was assessed using the modified Rankin Scale (mRS). 90-day mortality was recorded.

**Results:**

90 patients were analyzed (60 with, 30 without sepsis). Delirium occurred in 54.4% and structural brain injury in 42.2%. At ICU discharge, 23.3% had favorable neurological outcomes. NfL levels were higher in septic patients with delirium (p = 0.038). GFAP was significantly elevated in patients with structural brain injury (p < 0.001). All biomarkers showed prognostic potential; GFAP demonstrated the strongest association with unfavorable outcome (aOR 5.11, 95% CI 1.57–22.33). GFAP and UCH-L1 improved AUC in reference model 1 (age + SOFA), while all four biomarkers improved AUC in models 2 (age + GCS) and 3 (APACHE-II) for predicting poor outcome and 90-day mortality.

**Conclusion:**

Brain injury biomarkers correlate with delirium and structural injury and may enhance outcome prediction in heterogeneous critically ill patients.

**Trial registration:**

ClinicalTrials.gov. NCT06749483. Study Registration Date: 23 December 2024.

**Graphical abstract:**

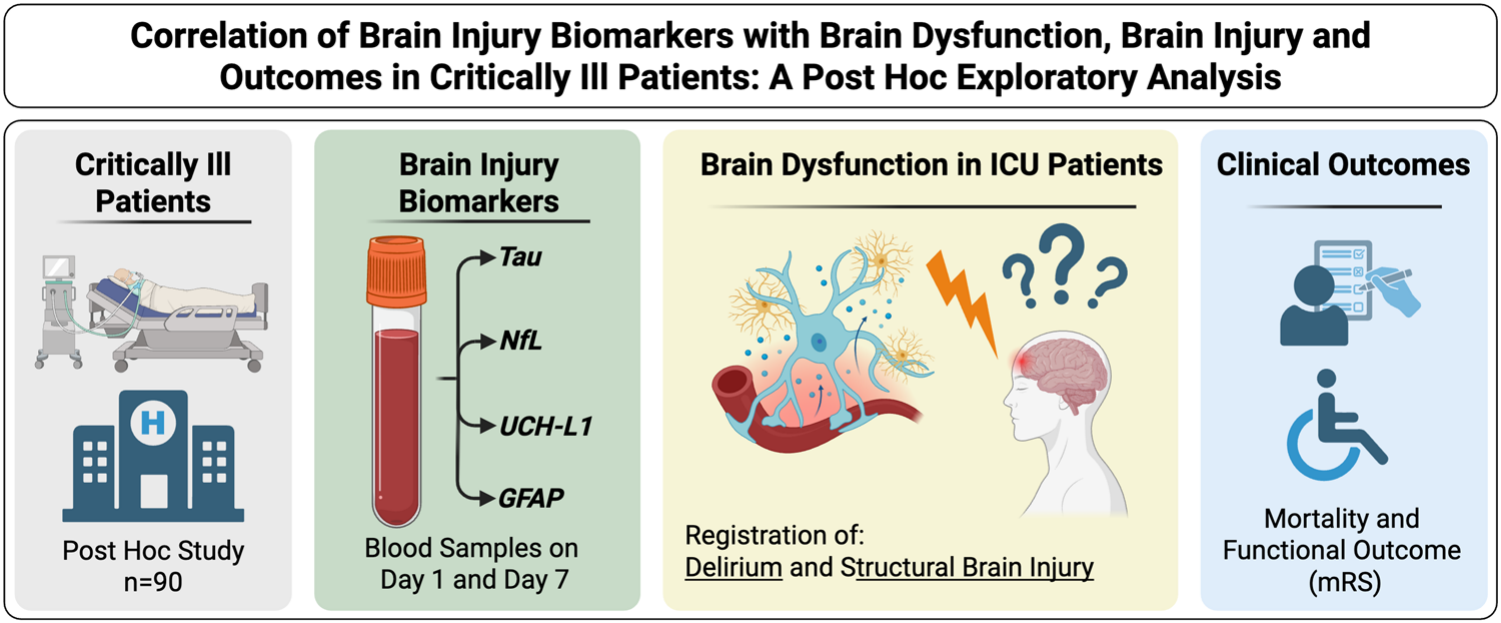

**Supplementary Information:**

The online version contains supplementary material available at 10.1007/s15010-026-02790-2.

## Background

In recent decades, efforts to improve outcomes following critical illness, whether due to severe infection or trauma, have intensified [1]. Although advances in critical care significantly reduced mortality, many patients still exhibit long-term neurological impairment [2–4]. Neurocognitive deficits frequently result from brain dysfunction due to sepsis-associated encephalopathy (SAE) or generally delirium of non-infectious origin, as well as structural brain injury in line with brain ischemia or hemorrhage [5–7], which can also complicate patients’ ICU stay [8]. Therefore, ICU patients are at high risk to develop long-term neurocognitive impairment [9].

Delirium is one of the most common neuropsychiatric complications in the ICU, occurs in 20–80% of cases, is frequently triggered by infection, trauma, or critical illness itself and contributes to increased morbidity and mortality [7, 10]. Particularly among those patients receiving sedation and mechanical ventilation, the detection of distinct clinical signs of brain dysfunction (i.e. delirium) or structural brain injury (i.e. ischemia or hemorrhage) is difficult to achieve [11–13]. In this acute phase of critical illness with a high percentage of unable-to-assess patients, ICU physicians would profit from a larger armamentarium of diagnostic instruments to first, detect brain dysfunction or injury and second, to optimize decision making which patient needs timely brain imaging in order to detect structural injury to the brain [14, 15]. While models such as IMPACT (International Mission for Prognosis and Clinical Trials in Traumatic Brain Injury) and CRASH (Corticosteroid Randomization after Significant Head Injury) [16, 17]combine parameters like age, GCS or pupil reactivity in order to predict outcomes after traumatic brain injury [18, 19], comparable validated tools for the assessment of delirium and subsequent brain injury in ICU patients are not available. Therefore, early and reliable diagnostic markers are warranted to identify patients at risk for brain dysfunction and brain injury and to guide necessary diagnostic procedures as well as treatment decisions to improve patient outcome [15, 20, 21]. Blood-based biomarkers of brain injury have emerged as promising tools to improve detection and risk stratification in this setting [21–23]. Glial fibrillary acidic protein (GFAP) reflects astroglial injury [24], while neurofilament light chain (NfL) is a marker of axonal damage [21]. Ubiquitin carboxy-terminal hydrolase L1 (UCH-L1) is predominantly expressed in neurons and indicates neuronal cell body injury [25], whereas total tau reflects microtubule disruption and neuronal degeneration [26]. These biomarkers have shown diagnostic and prognostic value in specific conditions such as traumatic brain injury [23], stroke, cardiac arrest and sepsis-associated encephalopathy [21, 27, 28], but their role in heterogeneous ICU populations, particularly in sepsis-associated brain dysfunction, remains less well defined.

Despite growing interest several gaps remain: First, most studies have focused on single biomarkers or highly selected populations, limiting generalizability to heterogeneous ICU cohorts [29]. Second, the relationship between these biomarkers and delirium or structural brain injury has not been consistently evaluated [30]. Third, whether these biomarkers may help differentiate between transient brain dysfunction and structural brain injury, and whether they may help predict neurological outcome, remains unclear [31]. However, recent studies suggest that brain injury biomarkers may offer incremental diagnostic and prognostic value for the prediction of long-term outcomes [23]in patients with TBI compared to pure clinical prediction models [32].

Therefore, the aim of this study involving a heterogeneous cohort of ICU patients with and without sepsis, was to explore whether biomarkers of brain injury are associated with brain dysfunction and structural brain injury, and whether they may provide additional diagnostic and prognostic information.

## Materials and methods

### Study design, ethical approval and trial registration

The present analysis was conducted as a post hoc analysis of the prospective observational MS-ICU study *“Gut microbiome – source of sepsis and novel target in intensive care units”,* a single-center cohort study at Jena University Hospital [33]. This study analyzed a heterogeneous cohort of critically ill patients with and without sepsis with presence or absence of primary brain injury and patients with or without secondary occurrence of brain injury during their ICU stay. Between January 2020 and August 2022, patients were prospectively enrolled. A total of 90 patients with available biomarker measurements and complete clinical data were included in the present analysis according to the initial study protocol. In accordance with the MS-ICU study protocol, blood samples were obtained at time point 1 (day of enrollment) and time point 2 (enrollment + 7 days). No off-schedule samples were recorded. We combined the prospectively acquired MS-ICU data with pre-defined medical data of the study participants derived from the digital patient management system. This retrospective data package included information on the past medical history, the clinical and neurological status including delirium assessments, neuroimaging parameters as well as data on morbidity and mortality during their ICU stay. The primary study as well as the post hoc analysis were approved by the local ethics board of the Friedrich-Schiller University Jena (Ethics identifier: 2019–1306 and 2024–3429-BO) and was registered (ClinicalTrials.gov: NCT06749483). The registration was performed after completion of the primary data collection and reflects the extended study protocol and analysis plan of the current investigation.

### Inclusion and exclusion criteria

Based on the MS-ICU study criteria, critically ill ICU patients ≥ 18 years of age with a severe infection receiving antibiotics, or those not on systemic antimicrobial therapy but expected to remain in the ICU for at least three days, were deemed eligible for the post hoc analysis.

MS-ICU exclusion criteria (based on microbiome analytics) comprised inflammatory bowel disease, a history of major bowel resection, selective decontamination of the oral and digestive tract, oral vancomycin therapy, immunocompromised patients, history of chemotherapy within the last 6 months, known travel history to countries of high antimicrobial resistance and refusal of study participation by the patient or a legal representative.

### Study visits and post hoc data collection

Data from standardized study visits were available from time point 1 and time point 2. Additionally, clinical, neurological, neuroimaging and outcome data with a focus on brain dysfunction and injury were assessed. The delirium assessment was performed using the Confusion Assessment Method-for the Intensive Care Unit (CAM-ICU) [11] as well as a patient chart review by an ICU physician (MR). Furthermore, the Glasgow Coma Scale (GCS) [34], the Richmond Agitation and Sedation Scale (RASS) [35], and the Sequential Organ Failure Assessment (SOFA) [36]score were used. Additionally, routine laboratory parameters, e.g. white blood cell count (WBC), C-reactive protein (CRP), procalcitonin (PCT), serum creatinine (creatinine), glomerular filtration rate (GFR), serum lactate (lactate) and blood glucose (glucose) were recorded.

Sepsis was diagnosed using SEPSIS-3 criteria [37].

Blood plasma samples from time point 1 and time point 2 were available for the measurement of blood-based brain injury biomarkers.

### Assessment of brain dysfunction, brain injury and neurological short-term outcome

Delirium was assessed using a combined approach including routine CAM-ICU screening and retrospective review of medical and nursing records (e.g. acute or fluctuating course of impaired consciousness and attention, disturbance of circadian rhythm, emotional dysregulation, global disturbance of cognition, psychomotor disturbance) including data on the pharmacological delirium treatment during the ICU stay [38]. The length of delirium was assessed by addition of all delirium-positive days. All available information was adjudicated by an ICU physician to determine final delirium status. In cases of discrepancy, classification was based on expert clinical judgment considering the overall clinical context.

Structural brain injury was assessed based on radiologically confirmed findings from routine cranial computed tomography (cCT) and/or magnetic resonance imaging (cMRI) performed during the study period. Brain injury was classified as primary brain injury, defined as acute intracranial pathology representing the principal cause of ICU admission (e.g., spontaneous intracerebral hemorrhage or large territorial ischemic stroke), or secondary brain injury, defined as new-onset intracranial pathology occurring in the course of the ICU stay (e.g., embolic ischemic stroke or hypoxic–ischemic injury following cardiac surgery).

The neurological status was assessed using the modified Rankin Scale (mRS) at the time of ICU admission and at discharge from hospital. A good or mild neurological impairment was defined by an mRS ≤ 3, while an mRS ≥ 4 was defined as poor neurological outcome.

### Sampling and analysis of blood-based biomarkers

Plasma samples were available from time point 1 (day of enrollment) and time point 2 (7 days after enrollment). Samples were immediately centrifuged at 2,000 × g for 15 min at 4° C, aliquoted, and stored at −80 °C until the time of analysis. Glial fibrillary acidic protein (GFAP), Neurofilament Light Chains (NfL), total tau protein (Tau) and Ubiquitin C-terminal hydrolase L1 (UCH-L1) were analyzed using Single-molecule arrays (Simoa, Neurology 4-Plex assay) on the HD-1 analyzer (Quanterix, Billerica, MA, USA) according to manufacturer’s instructions. Throughout the manuscript, ‘Tau’ refers to total tau protein. All biomarker samples were measured in duplicates. The analytical error was calculated as coefficient of variation (CV). For samples with a CV > 20% the measurement was repeated. No results were excluded. Missing biomarker data were primarily attributable to unavailable or insufficient plasma volumes at the time of analysis, as well as patient death prior to sample collection. An overview of missing samples is provided in the Supplementary Table (Table 1.2).

### Statistics

Descriptive statistics including means (standard deviation), medians (25th–75th percentile) were used for continuous variables as appropriate, whereas frequencies (percentages) were reported for binary or categorical variables to describe the patient cohort. Normal distribution was tested using Q–Q plots and the Shapiro–Wilk test. Log-transformation was used in highly skewed data. Continuous variables were compared by two-sided independent samples t test or Mann–Whitney U test between two groups, and two-sided Fisher’s exact test or χ^2^ test was applied for comparison of categorical variables. Linear regression models were applied to adjust for age and creatinine levels. Longitudinal changes from baseline in biomarker levels were assessed using the Wilcoxon rank-sum test and analysis of covariance (ANCOVA). Univariate and multivariate logistic regression models were used to assess the association between biomarker levels and patient outcomes, adjusting for age, renal function, and brain injury status. Odds ratios (ORs) with 95% confidence intervals (CIs) were reported. Subgroup analyses were conducted as follows: first, we compared brain injury biomarker levels in patients with and without delirium; second, we analyzed differences between patients with and without structural brain injury; third, we evaluated biomarker levels in septic and non-septic patients; and fourth, we assessed associations between biomarker values and patient outcomes.

Furthermore, discrimination was assessed using the area under the receiver operating characteristic curve (AUC). AUC values, 95% confidence intervals, p-values, and differences in AUC (ΔAUC) were estimated using bootstrap resampling with 2,000 iterations. Reference models —model 1 (Age + SOFA), model 2 (Age + GCS), and model 3 (APACHE II) [39] —were first constructed and subsequently extended by adding brain injury biomarkers to determine their incremental clinical value.

Analyses were restricted to patients with available biomarker data. Missingness differed between groups, as assessed by standardized mean differences for baseline characteristics (e.g., age and SOFA score), with a maximum value of 0.32.

Model analyses were conducted using complete-case data with patients grouped according to predefined clinical categories. A two-sided p value of less than 0.05 or a 95% CI not spanning zero was considered statistically significant.

All statistical analyses were performed using R version 4.2.2 (R Foundation for Statistical Computing, Vienna, Austria).

## Results

### Patient characteristics

**Fig. 1:**
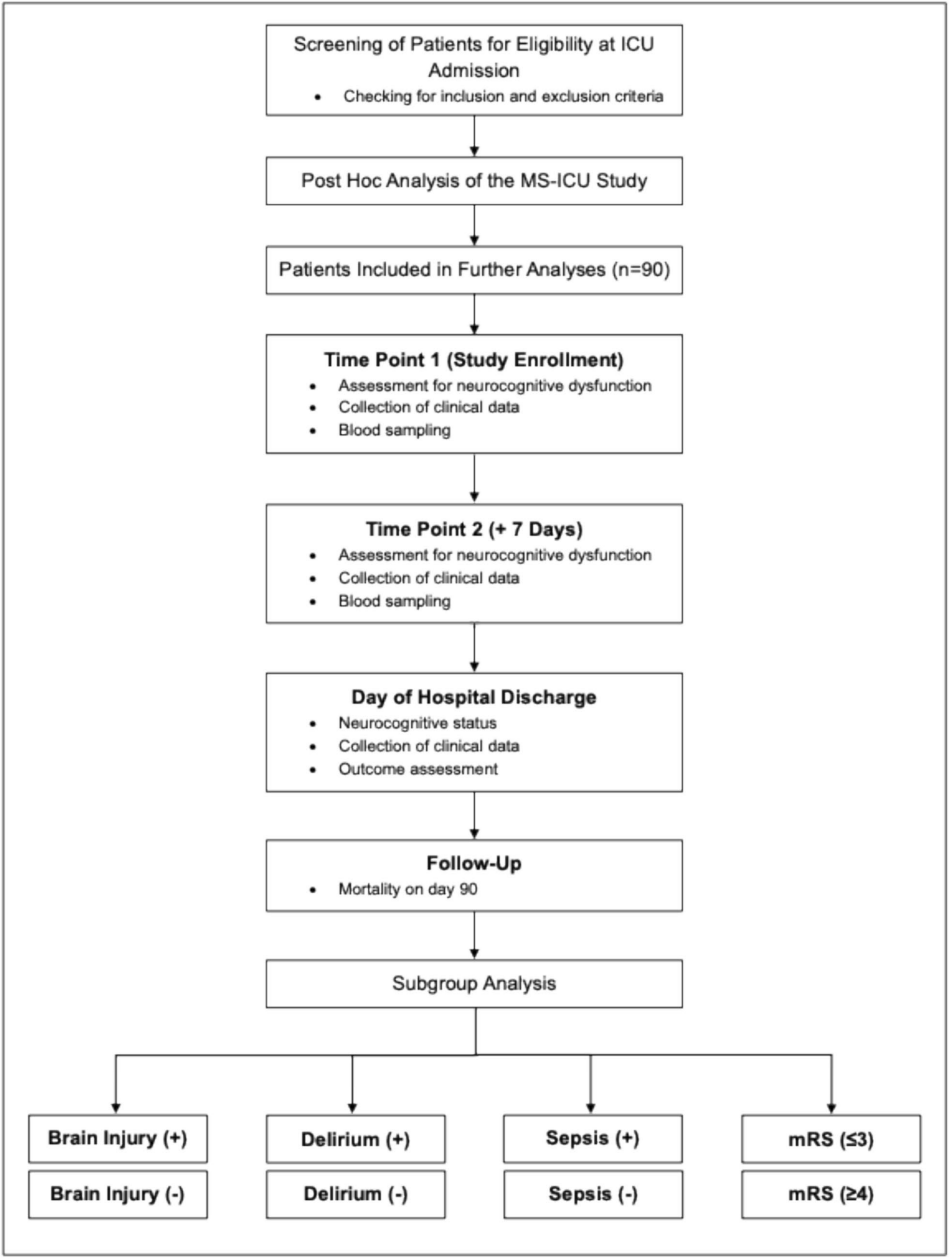
Study flowchart of patient selection

Between January 2020 and August 2022, a total of 90 patients were prospectively enrolled and were available for post hoc analysis (Fig. 1, Table 1). Patients had an average age of 64.2 ± 14.1 years, 32 of 90 (35.6%) were female and 58 of 90 (64.4%) were male. The overall body mass index (BMI) was 27.5 (23.9–31.5) kg/m^2^ and was not significantly different between the subgroups (Table 1). At time point 1, the median SOFA score was 7 (5–10), which significantly decreased to 6 (4–8) at time point 2 (p < 0.001). According to Sepsis-3 criteria, 60 out of 90 (66.7%) patients were septic during their ICU stay. The median length of ICU stay was 23.5 (17–30) days in the total cohort. Delirium occurred in 49 of 90 patients (54.4%), while structural brain injury was identified in 38 of 90 patients (42.2%) (Table 1).

**Table 1:** Baseline characteristics of patients

	Overall		Delirium		No Delirium			Brain Injury		No Brain Injury		
	N	Median (IQR)/%	N	Median (IQR)/%	N	Median (IQR)/%	p-value	N	Median (IQR)/%	N	Median (IQR)/%	p-value
*Demographics*												
Total	90	100	49	54.4	41	45.6	-	38	42.2	52	57.8	-
Age (Years)	90	64.2 (57,74)	49	67 (61,78)	41	64 (56,71)	0.059	38	59.5 (53,70.5)	52	68.5 (62.7, 78)	**0.001**
BMI	90	27.5 (23.9,31.5)	49	28.1 (24.7,31.6)	41	26.5 (23.3,30.5)	0.214	38	28.2 (24.1,31.4)	52	27.4 (23.7,31.5)	0.964
Sex (Female)	32	35.6	14	15.6	18	20	0.130	19	21.1	13	14.4	**0.014**
*Clinical Aspects*												
GCS Mild	22	24.4	11	12.2	11	12.2	0.165	3	3.3	19	21.1	**0.02**
GCS Moderate	19	21.1	14	15.6	5	5.6		7	7.8	12	13.3	
GCS Severe	49	54.4	24	26.7	25	27.8		28	31.1	21	23.3	
RASS	90	-3 (-4, -0.25)	49	-3 (-4,0)	41	-4 (-4,1)	0.202	38	-4 (-4.7, -2)	52	-2.5 (-4, 0)	**0.011**
SOFA Time Point 1	90	7 (5,10)	49	8 (6,10)	41	7 (5,9)	0.105	38	7 (5.2,9)	52	7 (4,10)	0.857
SOFA Time Point 2	90	6 (4, 8)	49	6 (3, 8)	41	6 (5, 8)	0.413	38	5 (3, 7)	52	7 (5, 10)	**0.007**
Emergency Admission	60	66.7	29	32.2	31	34.4	0.100	31	34.4	29	32.2	**0.01**
Preexisting neuropsychiatric disease	29	32.2	18	20	11	12.2	0.317	11	12.2	18	20	0.570
Extracorporeal Assist	42	46.7	29	32.2	13	14.4	**0.009**	12	13.3	30	33.3	**0.014**
Renal Replacement	27	30	18	20	9	10	0.127	9	10	18	20	0.265
Sepsis	60	66.7	38	42.2	22	24.4	**0.017**	20	22.2	40	44.4	**0.016**
Brain Injury	38	42.2	15	16.7	23	25.6	**0.015**	-	-	-	-	**-**
Delirium	49	54.4	-	-	-	-		15	16.7	34	37.8	**0.015**
*Outcomes*												
ICU Days	90	23.5 (17,30)	49	22 (17,30)	41	25 (17,30)	0.802	38	25 (20,30)	52	21.5 (11.8,30.3)	0.195
Hospital Days	90	31 (22.5,49)	49	31 (23,57)	41	31 (22,38)	0.408	38	32 (26,38.8)	52	30.5 (20.8,57.5)	0.794
Mortality on ICU	13	14.4	7	7.8	6	6.7	0.967	2	2.2	11	12.2	**0.037**
90-day Mortality	18	20.7	9	10.3	9	10.3	0.748	5	5.7	13	14.9	0.188
MRS before admission	90	2 (2,3)	49	2 (2,3)	41	2 (2,2)	0.449	38	2 (1,2)	52	2 (2,3)	0.089
MRS at discharge	90	5 (4,5)	49	5 (3,5)	41	5 (4,5)	0.713	38	5 (4,5)	52	4.5 (3,5)	**0.01**
*Laboratory*												
WBC (Gpt/l)	90	12.7 (8.7,16.4)	49	13.1 (8.6,16.8)	41	12.1 (9,14.5)	0.463	38	11.4 (8.3,14.1)	52	13.3 (9.7,18.5)	0.07
CRP (mg/l)	90	106.1 (59.8,184.7)	49	107.3 (59.8,178.8)	41	89.70 (58.8,186.7)	0.644	38	78.5 (53.5,106.9)	52	137 (63.1,219)	**0.002**
PCT (ng/ml)	89	0.48 (0.17,1.5)	49	0.73 (0.37,1.7)	40	0.23 (0.09,0.7)	**0.001**	37	0.22 (0.09,0.64)	52	0.74 (0.36,2.2)	**0.01**
Creatinine (µmol/l)	90	96 (56,141.7)	49	122 (72,191)	41	73 (51,108)	** < 0.001**	38	67 (49.3,109.5)	52	120 (77,158.8)	** < 0.01**
GFR (ml/min)	89	64.4 (36.3,102.5)	48	45.9 (29.6,89.2)	41	88.2 (51.1,111.8)	**0.004**	38	95.6 (51.4,114.8)	51	46.5 (35.3,86.8)	**0.02**
Lactate (mmol/l)	90	1 (0.8,1.2)	49	1.00 (0.8,1.3)	41	1.00 (0.8,1.2)	0.224	38	0.95 (0.7,1.2)	52	1.0 (0.8,1.2)	0.187
Glucose (mmol/l)	90	7.9 (6.9,8.8)	49	8.2 (6.9,9.2)	41	7.9 (6.6,8.4)	0.089	38	7.9 (6.9,8.8)	52	8 (6.9,8.9)	0.753
Blood-based Brain Injury Biomarker Time Point 1												
GFAP (pg/ml)	61	215.1 (136.4–288.4)	37	234.9 (136.5–367.1)	24	185.5 (131.5–267.2)	0.396	11	404.4 (161.9 852.9)	50	208.9 (126.6–268.0)	**0.046**
NfL (pg/ml)	59	224.6 (85.0–467.0)	36	255.5 (109.8–589.7)	23	132.9 (80.6–337.8)	0.104	13	130.1 (72.1–510.6)	46	230.8 (103.0–419.9)	0.891
Tau (pg/ml)	73	6.9 (3.7–11.6)	43	8.7 (3.7–16.4)	30	6.2 (3.5–10.5)	0.265	22	6.2 (3.7–11.3)	51	7.8 (3.7–13.9)	0.723
UCH-L1 (pg/ml)	73	49.5 (28.3–98.3)	43	48.3 (25.6–109.2)	30	56.2 (28.3–98.3)	0.969	22	89.9 (31.4–116.9)	34	48.2 (24.6–90.2)	0.080
Blood-based Brain Injury Biomarker Time Point 2												
GFAP (pg/ml)	60	227.7 (129.1–516.4)	34	212.5 (114.4–441.9)	26	241.3 (157.4–605.8)	0.254	26	396.1 (207.4–1510.3)	34	194.4 (122.7–286.2)	**0.012**
NfL (pg/ml)	44	426.4 (235.7–787.0)	27	552.5 (281.8–925.9)	17	335.0 (178.7–657.8)	0.162	14	425.4 (165.7–832.8)	30	426.4 (281.8–783.9)	0.696
Tau (pg/ml)	61	6.5 (4.1–11.1)	35	5.7 (2.8–12.0)	26	7.5 (4.3–9.3)	0.526	27	7.3 (4.1–12.0)	51	5.7 (3.0–11.1)	0.412
UCH-L1 (pg/ml)	61	74.8 (46.9–116.2)	35	84.1 (56.0–123.0)	26	64.7 (45.2–116.2)	0.605	27	89.6 (53.2–169.4)	34	70.0 (45.2–102.9)	0.267

Among the 90 patients, 21 out of 90 (23.3%) had a favorable short-term neurological outcome (mRS ≤ 3), whereas 69 out of 90 (76.7%) developed severe neurological impairment (mRS ≥ 4). The mortality rate of the total cohort until the end of the ICU stay was 14.4% (13 of 90 patients). From the delirium group 7 out of 49 patients (14.3%) died during the ICU stay, compared to 6 out of 41 patients (14.6%) without delirium. Two out of 38 patients (5.3%) with structural brain injury died, compared to 11 out of 52 patients (21.2%) without brain injury. (Table 1).

### Longitudinal course of brain injury biomarkers

Within the total cohort of ICU patients, we observed a significant increase of NfL levels between time point 1 and time point 2 [224.6 (85.0–467.0) pg/mL vs. 426.4 (235.7–787.0) pg/mL; p < 0.001]. Tau values showed a significant decrease between time point 1 and time point 2 [6.9 (3.7–11.6) pg/mL vs. 6.5 (4.1–11.1) pg/mL; p = 0.002]. GFAP [215.1 (136.4–288.4) pg/mL vs. 227.7 (129.1–516.4) pg/mL; p = 0.127] and UCH-L1 [49.5 (28.3–98.3) pg/mL vs. 74.8 (46.9–116.2) pg/mL; p = 0.188] did not show significant longitudinal changes in the total cohort.

To further assess the diagnostic value of brain injury biomarker levels, we conducted detailed subgroup analyses. (Fig. 1).

### Brain injury biomarker levels in patients with and without delirium

Delirium was present in 49 out of 90 patients (54.4%), with a median delirium duration of 7.0 (5.0–10.8) days. Hyperactive delirium was present in 25 out of 49 patients (51.0%), mixed in 16 (32.7%), and hypoactive in 8 (16.3%).

Pharmacological delirium therapy comprised α2-agonists (clonidine), high-potency neuroleptics (haloperidol), low-potency neuroleptics (pipamperone), and atypical neuroleptics (risperidone).

**Fig. 2:**
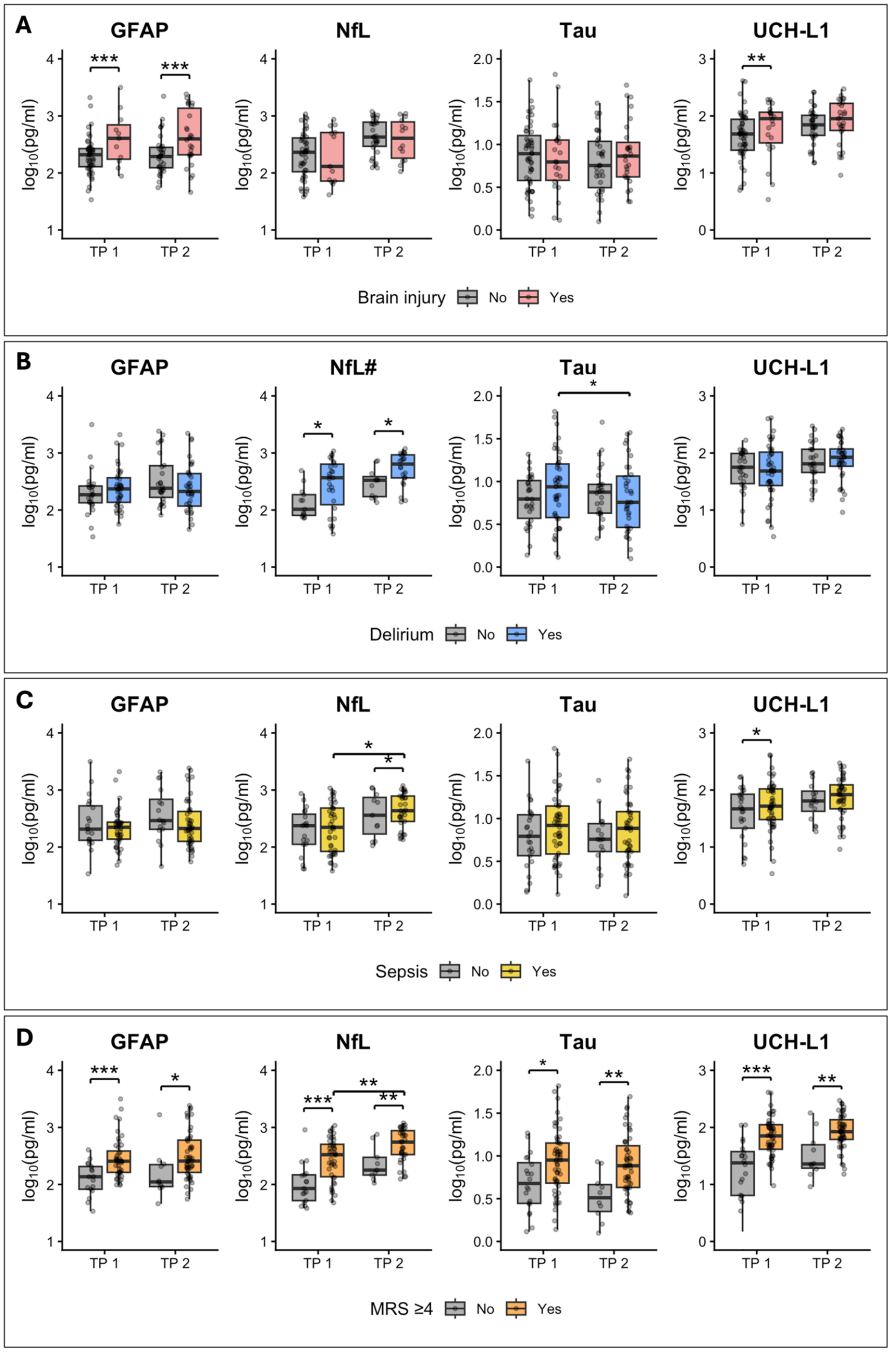
Box Plot of Biomarkers. Longitudinal course of brain injury biomarkers (UCH-L1, ubiquitin carboxyl-terminal hydrolase L1; GFAP, glial fibrillary acidic protein; NfL, neurofilament light chain; Tau, total tau protein) in different subgroups of the study. **A** Structural Brain Injury (BI), **B** Delirium, **C** Sepsis, **D** Functional Outcome represented by modified Rankin Scale (mRS); # Delirium Assessment in Patients with Sepsis; *TP* Time Point

At enrollment, patients with and without delirium were comparable in terms of age, sex, and SOFA score (all p > 0.05; Table 1). However, patients with delirium had significantly higher creatinine levels [122 (72–191) vs. 73 (51–108) µmol/L; p < 0.001] and lower GFR [45.9 (29.6–89.2) vs. 88.2 (51.1–111.8) mL/min; p = 0.004]. NfL levels increased significantly over time in both delirium-positive and delirium-negative patients (both p ≤ 0.002). GFAP, Tau, and UCH-L1 did not show consistent longitudinal differences between groups as shown in Fig. 2.

### Brain injury biomarker levels in patients with and without structural brain injury

Structural brain injury was present in 38 (42.2%), no brain injury in 52 of 90 ICU patients (57.8%). Among those with structural brain injury, 25 (27.8% of the total cohort) were classified as primary brain injured and 13 (14.4%) developed secondary brain injury during ICU treatment. This heterogeneous subgroup includes both patients with predominantly hemorrhagic pathologies (21 (55.3%)) as well as those with predominantly ischemic conditions 17 of 38 patients (44.7%).

Compared to those without brain injury, patients with brain injury were younger and had better renal function (Table 1). Biomarker concentrations in patients with and without structural brain injury are shown in Fig. 2. GFAP levels were significantly elevated in patients with brain injury at time point 1 [404.4 (161.9–852.9) vs. 208.9 (126.6–268.0) pg/mL; p < 0.001] and time point 2 [396.1 (207.4–1510.3) vs. 194.4 (122.7–286.2) pg/mL) pg/mL; p < 0.001]. Furthermore, UCH-L1 levels were significantly elevated at time point 1 [89.9 (31.4–116.9) vs. 48.2 (24.6–90.2) pg/mL; p < 0.001]. This difference remained statistically significant after adjusting for age and creatinine. (Fig. 2).

### Brain injury biomarker levels in patients with and without sepsis

In this cohort of critically ill patients, 60 out of 90 (66.7%) fulfilled Sepsis-3 criteria. Compared to non-septic patients, those with sepsis were significantly sicker, as indicated by higher SOFA scores (9 [7–10] vs. 4 [2.3–6], p < 0.001), and had a significantly higher mortality (13 [21.7%] vs. 0 [0%], p = 0.006). Biomarker levels were elevated in septic patients, with longitudinal NfL values remaining persistently elevated (Supplemental Table 2.3). By comparison of patients with and without sepsis, NfL levels increased significantly over time in both groups. However, after excluding patients with brain injury status, this upward trajectory was observed only in septic patients as shown in ANCOVA analyses adjusting time point 2 NfL levels for baseline concentrations. NfL remained higher in septic patients (445.6 [376–531] pg/mL vs. 270.5 [175–417] pg/mL; ratio 1.65, 95% CI 1.05–2.59; p = 0.038). Further we found significantly higher NfL levels in septic patients with delirium compared to those without delirium at time point 1 [368.2 (107.3–668.9) pg/mL vs. 103.0 (80.6–186.0) pg/mL; p < 0.039] and time point 2 [644.1 (366.0–925.9) pg/mL vs. 332.5 (165.7–433.8) pg/mL; p < 0.014], even after adjusting for age. Furthermore, septic patients showed significantly higher UCH-L1 age-adjusted values at time point 1 [49.5 (31.0–95.9) pg/mL vs. 25.8 (8.7–62.5) pg/mL; p = 0.012] and higher age-adjusted NfL values at time point 2 [504.7 (335.0–790.0) pg/mL vs. 177.9 (123.2–296.3) pg/mL; p = 0.022], compared to non-septic patients (Supplemental Table 3.3.1).

### Correlation of brain injury biomarker levels with outcome parameters

All biomarkers were associated with worse neurological status and showed higher levels in patients with an mRS Score greater than 4. (Supplemental Table 3.4; Table 3.4.1).

GFAP, NfL, Tau and UCH-L1 showed significantly elevated blood levels at time point 1 and time point 2 in patients with poor neurological outcome, as presented in Fig. 2. These associations remained significant for GFAP, NfL and UCH-L1 after adjusting for age and renal function. (Supplemental Table 3.4; Table 3.4.1).

Further subgroup analysis revealed that patients with mRS ≥ 4 at discharge experienced a notable rise in longitudinal NfL levels [332.4 (132.9–510.6) pg/mL vs. 552.5 (332.5–922.1) pg/mL; p < 0.001], while those with favorable outcome (mRS ≤ 3) did not show a significant change over time [85.0 (52.1–147.0) pg/mL vs. 178.7 (146.0–298.4) pg/mL; p = 0.141] (Supplemental Table 2.5). After exclusion of patients with structural brain injury, ANCOVA analyses adjusting for baseline concentrations showed higher time point 2 NfL levels in patients with mRS ≥ 4 (463.1 [389.2–550.6] pg/mL vs. 279.2 [198.7–392.3] pg/mL; ratio 1.66, 95% CI 1.15–2.40; p = 0.013).

Univariate logistic regression analysis showed that elevated levels of each biomarker were significantly associated with increased odds of unfavorable outcomes. GFAP showed an odds ratio (OR) of 6.6 (95% CI: 2.27–26.51), UCH-L1 had an OR of 4.59 (95% CI: 2.26–11.67), NfL demonstrated an OR of 3.78 (95% CI: 1.82–9.26), and Tau was associated with an OR of 2.37 (95% CI: 1.22–5.04) (Supplementary Table 5.1).

In multiple logistic regression analyses adjusted for brain injury, age and renal function, three out of four elevated biomarker levels remained associated with unfavorable outcomes. GFAP showed an adjusted odds ratio (aOR) of 5.11 (95% CI: 1.57–22.33), UCH-L1 had an aOR of 4.17 (95% CI: 1.9–11.35), NfL demonstrated an aOR of 3.87 (95% CI: 1.57–12.05), and Tau was not associated (Supplementary Table 5.2).

To further explore the additional prognostic value of brain injury biomarkers, we used reference model 1 (Age + SOFA), 2 (Age + GCS) and 3 (APACHE II) as basic models to clinically predict patient outcome.

The area under the curve (AUC) for reference model 1 was 0.841 (95% CI: 0.742–0.930) to predict an mRS ≥ 4, an AUC of 0.814 (95% CI: 0.707–0.905) to predict ICU mortality and an AUC of 0.756 (95% CI: 0.639–0.862) to predict 90-day mortality. For reference model 2, the AUC was 0.869 (95% CI: 0.770–0.944) for an mRS ≥ 4; 0.751 (95% CI: 0.609–0.873) for ICU mortality and 0.705 (95% CI: 0.579–0.823) to predict 90-day mortality. For reference model 3, the AUC was 0.760 (95% CI: 0.652–0.855) for an mRS ≥ 4; 0.857 (95% CI: 0.732–0.949) for ICU mortality and 0.821 (95% CI: 0.715–0.911) to predict 90-day mortality.

**Fig. 3:**
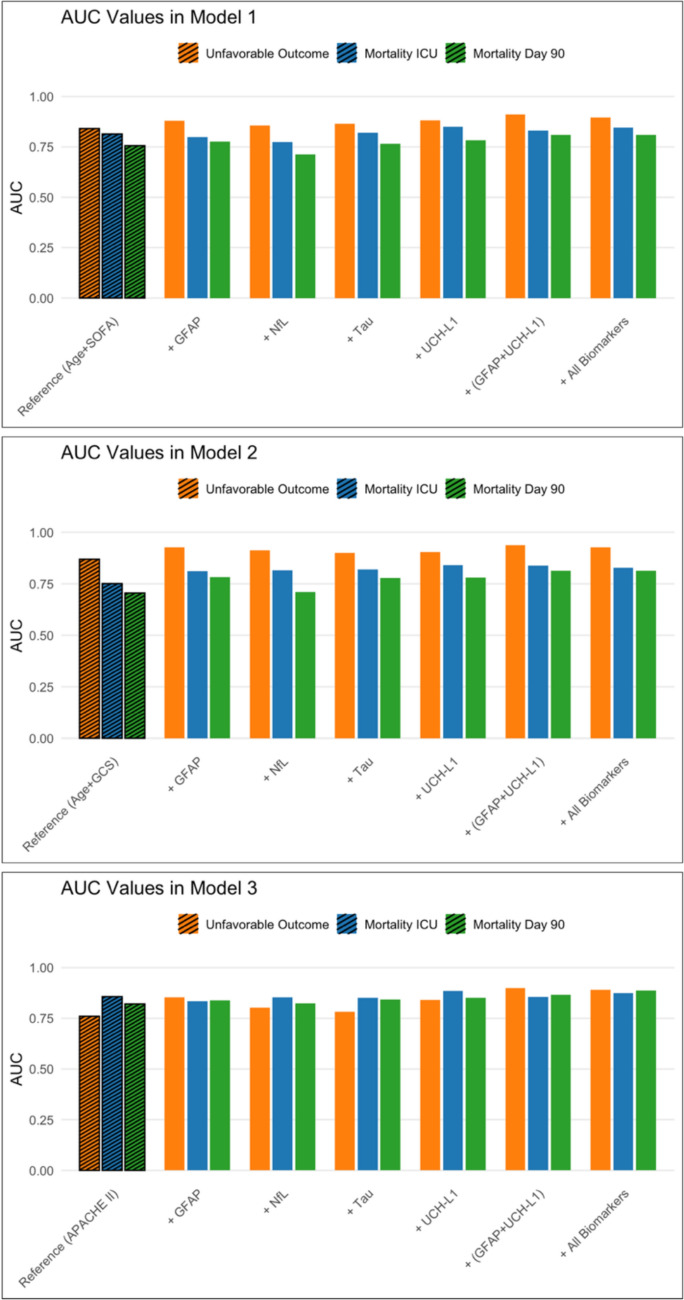
AUC Values. AUC values for Model 1 (Age + SOFA), Model 2 (Age + GCS) and Model 3 (APACHE II) are presented, with the reference model shown alongside the corresponding models incorporating biomarkers.

Addition of biomarkers resulted in overall increases in AUC values across all models (Fig. 3; Supplemental Tables 4.1–4.3). The inclusion of GFAP and UCH-L1 together significantly improved model 1 for predicting mRS ≥ 4 (ΔAUC 0.057 [0.013–0.191]). In model 2, adding all four biomarkers also yielded a significant improvement (ΔAUC 0.024 [0.003–0.142]).

**Fig. 4:**
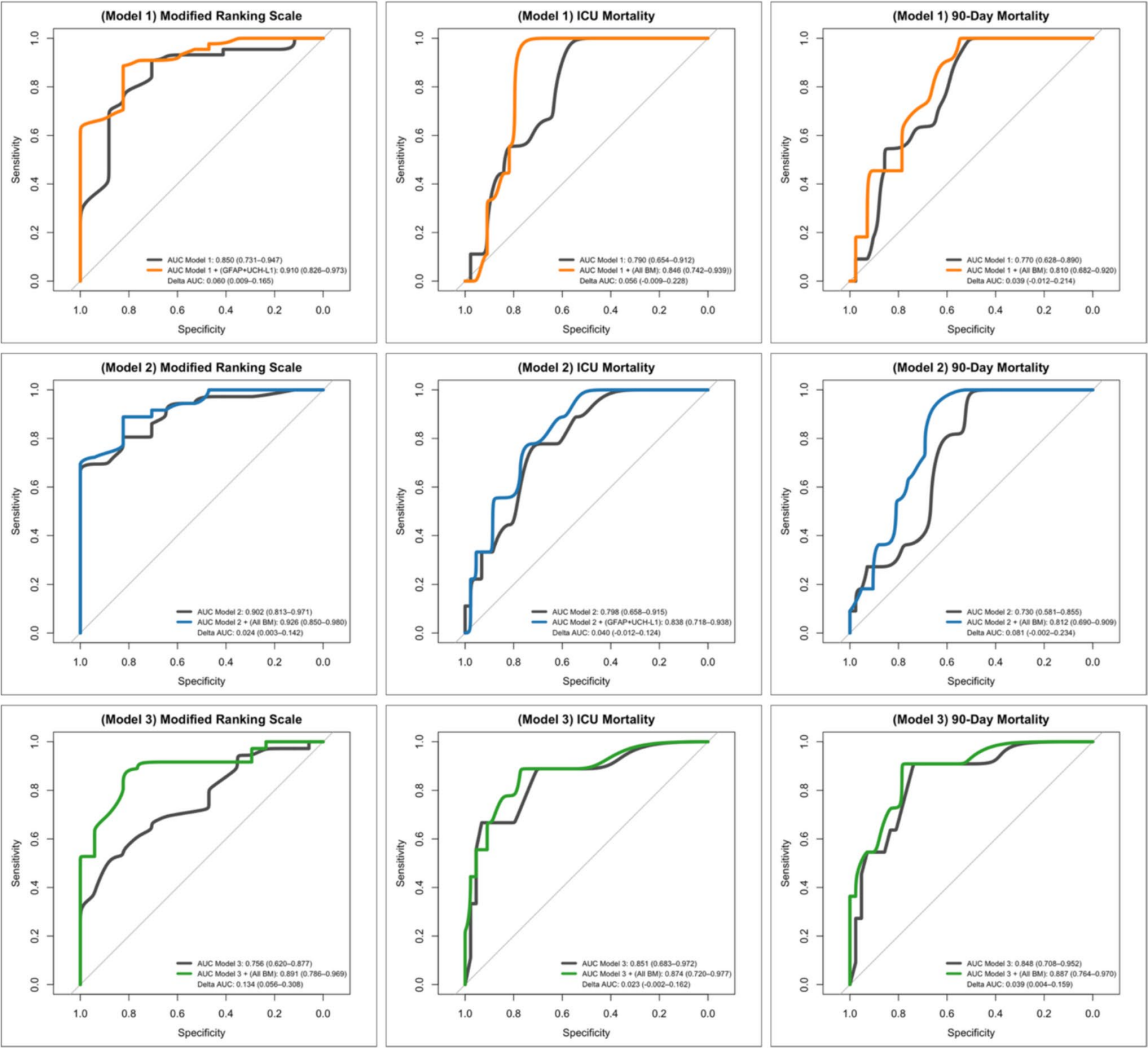
ROC Curve. ROC Curve Analysis of the Basic Clinical Model With and Without Brain Injury Biomarker. Model 1 (Age + SOFA); Model 2 (Age + GCS); Model 3 (APACHE II). The grey line represents the reference model, and the colored lines represent the reference model combined with the best-performing biomarker or biomarker combination

Model 3 demonstrated further improvement with the addition of all four biomarkers, both for predicting mRS ≥ 4 (ΔAUC 0.134 [0.056–0.308]) and 90-day mortality (ΔAUC 0.039 [0.004–0.159]) (Supplemental Tables 4.1–4.3; Fig. 4; Supplemental Figure 1).

## Discussion

In the present heterogeneous cohort of ICU patients, the integration of a panel of blood-based brain injury biomarkers revealed potential additional diagnostic value in daily clinical practice. Our findings demonstrate that these four different biomarkers may improve the detection of brain injury and delirium in critically ill individuals and may provide additional prognostic insights into short-term neurological outcomes and mortality following critical illness.

Similar findings have been reported in previous studies, including narrative and systematic reviews [29, 40–42], as well as research on delirium in ICU cohorts [30, 43, 44], sepsis, critically ill patients, and traumatic brain injury [45, 46]. Although these studies often focus on specific subgroups with stricter inclusion and exclusion criteria, they support the consistency of our results. In contrast, our study demonstrates the broader applicability of these distinct biomarkers in rather unselected heterogeneous ICU cohorts.

While biomarker-based approaches in critical care research have focused on five pathophysiological processes such as inflammation, endothelial dysfunction, coagulopathy, immune dysregulation, and endotoxemia—as recently reported by Nynatten Van et al. [47]- biomarkers of brain injury have been less frequently included, despite their intrinsic relevance for clinical outcomes, as shown in the present study.

We found that NfL levels were higher in septic patients with delirium compared to those without delirium. Furthermore, GFAP levels were significantly higher in patients with structural brain injury compared with those without, indicating an association with structural brain injury. In further statistical analyses we found that confounding factors such as brain injury, age and renal function have major impact on the interpretation of the results. Biomarker-guided assessment may offer additional prognostic information beyond established clinical risk factors; however, existing evidence supporting incremental value over ICU-specific severity scores such as age, GCS, SOFA, and APACHE II remains limited [23, 32]. Higher age, higher SOFA and lower GCS have shown associations with higher delirium rates and worse neurological outcome [48, 49], whereas APACHE II is a standardized ICU Benchmark Score [39].

In this present exploratory analysis using a mixed cohort of ICU patients, we showed an improved outcome prediction using brain injury biomarkers. The SOFA score and age derived from CRASH can form a solid reference model for outcome prediction because both are strong, well-established predictors of patient mortality and disease severity in critically ill patients [36, 50, 51]. Age reflects the patient’s baseline physiological reserve and vulnerability [51], while the SOFA score quantifies the extent of organ dysfunction during critical illness [36]. The GCS is a strong predictor of patient outcomes because it objectively measures the level of consciousness and neurological function, although it is partially dependent on the administration of sedatives. Lower GCS scores indicate more severe brain injury or dysfunction, which directly correlates with higher mortality and worse prognosis. Therefore, GCS serves as a simple and reliable indicator of disease severity in outcome prediction models [52]. Together, Age, SOFA and GCS may serve as a simple yet robust benchmark against which more complex predictive models can be compared [52–54].

The overall delirium prevalence was 54.4% (49/90 patients), which is comparable to recent data [10, 43, 55]. Brain injury occurred in 42.2% (38/90 patients) which may be explained by the heterogeneity of the ICU cohort, including patients with primary brain injury, comprising stroke, ischemia, and post-neurosurgical intervention.

As noted earlier, NfL appears promising for a range of diagnostic applications. They have been extensively studied over the past decades as a marker of neuronal injury, with emerging evidence suggesting associations with delirium and critical illness in adults [21, 31, 56–58]. NfL increase is often associated with systemic inflammation, which can trigger neuroinflammation and ultimately lead to axonal injury [21, 57].

It is well-established that independently from specific diseases, NfL levels increase with age and that age is a strong predictor of intensive care outcomes in critically ill patients [51, 59]. Furthermore, poor renal function results in elevated NfL levels. Therefore, we included these confounding factors age and creatinine levels in our statistical analyses, revealing that NfL was still significantly higher in patients with unfavorable neurological outcome. Overall these biomarkers, have certain limitations due to confounding factors, (e.g. age, renal function, and pre-existing neurological comorbidities) [21], which should be considered when interpreting results.

NfL levels increased over time during ICU stay, particularly in patients with sepsis and in those with unfavorable outcome, whereas other biomarkers did not show consistent changes over time, which may be related to evolving neurological impairment. Importantly, the association between higher NfL levels and both sepsis and unfavorable neurological outcome remained evident after adjustment for baseline concentrations, supporting an association beyond initial differences at ICU admission. Mechanisms such as cerebral hypoperfusion, hypoxemia, metabolic derangements, systemic inflammation, and blood–brain barrier disruption are thought to contribute to the release of intracellular proteins (e.g., Tau, GFAP, NfL, UCH-L1) into the bloodstream, potentially exacerbated by systemic insults including hypotension, hypoxemia, inflammation, and endothelial dysfunction [60].

Biomarkers of brain injury are promising adjunctive diagnostic tools to deeper evaluate patients in a critical care environment. They may help to detect neurocognitive impairment and emerging brain injury in patients without reliable neurological assessment. This would enable clinicians to detect structural brain injury earlier during the ICU stay and to identify patients to profit from neurocognitive rehabilitation following the ICU. Taken together, biomarker profiles may help differentiate predominant astroglial injury (GFAP) from neuronal/axonal injury (NfL, Tau) and acute neuronal cell-body injury (UCH-L1), with concurrent elevations suggesting a mixed injury. Accordingly, a panel combining GFAP, NfL, Tau, and UCH-L1 may more comprehensively capture distinct yet overlapping patterns of brain injury than any single biomarker alone. However, it is important to note that the four analyzed biomarkers reflect distinct patterns and temporal trajectories. For example, NfL levels increased longitudinally in septic patients, whereas tau levels decreased in patients with delirium. This divergence may reflect differences in underlying pathophysiology as well as the influence of confounding factors and timing of measurement. In particular, the absence of baseline values may limit the ability to fully characterize individual variability and biomarker dynamics over time.

Assessment of delirium is particularly difficult in ICU patients due to sedation and mechanical ventilation. Krewulak et al. analyzed a chart-based delirium detection tool to be adapted and validated in ICU adults, and how it performs alone or alongside classical routine ICU screening tests like the CAM-ICU and ICDSC. They found that it showed good reliability and moderate accuracy (sensitivity 66%, specificity 82%) alone, which improved with a combination using routine screening tools (CAM-ICU or ICDSC), raising the AUC to approximately 80%. Key chart cues linked to delirium included confusion, disorientation, fluctuating RASS scores, hallucinations, and antipsychotic use. False negatives occurred when symptoms weren’t documented, while false positives often came from unspecific documentation. Overall, they concluded that it is best used alongside routine screening or for retrospective research, not as a stand-alone diagnostic tool [38], as performed in the present study.

### Strength and limitations of our study

This study has several important methodological strengths. Data were acquired prospectively, ensuring systematic and consistent collection of clinical and laboratory variables. Additionally, a structured post hoc analysis was conducted using data from the patient data management system. Standardized chart reviews using predefined neurological and delirium parameters of the neurological data on delirium and outcome beyond prospective CAM-ICU assessment and enabled us to correlate biomarker data with distinct clinical endpoints. This method has been proven to be effective in a recent study testing the adaptation and validation of a Chart-Based Delirium Detection Tool for the ICU [38].The panel of four brain injury biomarkers used in this study enabled us to assess multiple compartments within the central nervous system, including neuronal (UCH-L1), axonal (NfL, Tau), and glial compartments (GFAP). Biomarker measurements were performed at predefined time points, allowing for a structured assessment of temporal dynamics and reducing variability related to sampling. In addition, the ICU cohort was comprehensively characterized, with detailed clinical, physiological, and outcome data. This assessment provided insights into treatment efficacy and revealing the extent of delirium and neurocognitive impairment.

The present study has several limitations. It was conducted as a single-center trial, which may limit the generalizability of the findings to other ICU settings with different patient populations. This exploratory, hypothesis-generating study intentionally included a heterogeneous, neurologically unselected ICU cohort to reflect real-world clinical practice and to demonstrate the feasibility of blood-based biomarker panels in this setting. In contrast to previous biomarker studies aimed at validation and cut-off determination, which employed more restrictive inclusion and exclusion criteria, our cohort was defined more broadly. While this approach enhances clinical applicability, it may introduce greater variability, residual confounding, and potential effect dilution. Therefore, comparability with more selected homogeneous cohorts is limited and further large-scale studies are needed to confirm our results.

Future research should incorporate high-frequent repeated biomarker measurements, sex-stratified analysis and include baseline measurement to improve temporal resolution. This would be ideal for an individualized biomarker-guided patient assessment, given the interindividual variability of biomarker measurements. These studies should also include dedicated long-term follow-up assessments to better characterize patient trajectories after critical illness.

This exploratory analysis was restricted to patients with available biomarker data. Missing biomarker data (Supplementary Table 1.2), particularly at time point 2, were mainly due to ICU discharge, death, or logistical constraints. Analyses at this time point were therefore based on a reduced sample size. Given the differential missingness between groups, informative missingness and potential selection bias cannot be fully excluded. Accordingly, the findings should be interpreted as hypothesis-generating. Furthermore, the inclusion of multiple biomarkers relative to the number of outcome events increases the risk of overfitting, which may limit the model’s generalizability to external populations.

## Conclusions

Biomarkers of brain injury significantly increase over time in heterogeneous cohorts of critically ill septic or non-septic ICU patients and correlate with delirium, structural brain injury and short-term patient outcome. Sepsis is a strong trigger of brain dysfunction associated with increased biomarker levels. Validated panels of brain injury biomarkers may provide additional clinical and prognostic value in critically ill patients. Their potential use to differentiate transient brain dysfunction from structural injury requires further investigation. Therefore, these exploratory findings need external validation and confirmation in adequately powered prospective studies to judge their diagnostic utility.

## Supplementary Information

Below is the link to the electronic supplementary material.

Supplementary file1 (DOCX 405 kb)

Supplementary file2 (PDF 202 kb)

## Data Availability

The data underlying this article are available in the article and in its online supplementary material. Request for additional information can be made to the corresponding author.

## Abbreviations

α2-agonists: Alpha-2 adrenergic receptor agonists
aOR: Adjusted odds ratio
AUC: Area under the curve
BI: Brain injury
BMI: Body mass index
BM: Biomarker
cCT: Cranial computed tomography
CI: Confidence interval
cMRI: Cranial magnetic resonance imaging
CRASH: Corticosteroid Randomisation After Significant Head Injury
CRP: C-reactive protein
CV: Coefficient of variation
GCS: Glasgow Coma Scale
GFAP: Glial fibrillary acidic protein
GFR: Glomerular filtration rate
ICDSC: Intensive Care Delirium Screening Checklist
ICU: Intensive care unit
IMPACT: International Mission for Prognosis and Analysis of Clinical Trials in Traumatic Brain Injury
IQR: Interquartile range
mRS: Modified Rankin Scale
MS-ICU: “Gut microbiome – source of sepsis and novel target in intensive care units”
NfL: Neurofilament light chain
OR: Odds ratio
PCT: Procalcitonin
RASS: Richmond Agitation–Sedation Scale
ROC: Receiver operating characteristic
SAE: Sepsis-associated encephalopathy
SEPSIS-3: Third International Consensus Definitions for Sepsis and Septic Shock
Simoa: Single-molecule array
SMD: Standardized Mean Difference
SOFA: Sequential Organ Failure Assessment
TBI: Traumatic brain injury
UCH-L1: Ubiquitin carboxyl-terminal hydrolase L1
WBC: White blood cell count
