# Supplementary material for: Correlation of brain injury biomarkers with brain dysfunction, brain injury, and outcomes in critically ill patients: a post hoc exploratory analysis
Source: Infection. 2026 Apr 13;54(3):1501–17. doi: 10.1007/s15010-026-02790-2 (PMC13323603; doi:10.1007/s15010-026-02790-2)
Supplement: Supplementary file 1 — Supplementary file1 (DOCX 405 kb) [file 15010_2026_2790_MOESM1_ESM.docx]

**Supplementary Tables**

[Figure 1 Delta AUC Values 3](#_Toc223444971)

[Table 1.1 - Comorbidities 4](#_Toc223444972)

[Table 1.2 - Missing Biomarker Data 4](#_Toc223444973)

[Table 2.1 - Longitudinal Biomarker Assessment (Wilcoxon Signed-Rank Test); Entire Cohort 5](#_Toc223444974)

[Table 2.2 - Longitudinal Biomarker Assessment (Wilcoxon Signed-Rank Test); Subgroup Brain Injury 5](#_Toc223444975)

[Table 2.2.1 - Analysis of Covariance by Brain Injury Status (Yes/No) 5](#_Toc223444976)

[Table 2.3 - Longitudinal Biomarker Assessment (Wilcoxon Signed-Rank Test); Subgroup Delirium 5](#_Toc223444977)

[Table 2.3.1 - Analysis of Covariance by Delirium Status (Yes/No) 6](#_Toc223444978)

[Table 2.4 - Longitudinal Biomarker Assessment (Wilcoxon Signed-Rank Test); Subgroup Sepsis 6](#_Toc223444979)

[Table 2.4.1 - Analysis of Covariance by Sepsis Status (Yes/No) 7](#_Toc223444980)

[Table 2.5 - Longitudinal Biomarker Assessment (Wilcoxon Signed-Rank Test); Subgroup mRS 7](#_Toc223444981)

[Table 2.5.1 - Analysis of Covariance by mRS Status (mRS ≥4/ mRS ≤3) 8](#_Toc223444982)

[Table 2.5.2 - Analysis of Covariance by mRS Status (mRS ≥4/ mRS ≤3), Brain Injury excluded 8](#_Toc223444983)

[Table 3.1 - Biomarker Assessment (Mann–Whitney U Test) on Day 1 and Day 7 – Brain Injury Subgroup 9](#_Toc223444984)

[Table 3.1.1 - Linear Regression Models Adjusted for Age and Age + Creatinine 9](#_Toc223444985)

[Table 3.2 - Biomarker Assessment (Mann–Whitney U Test) on Day 1 and Day 7 – Delirium Subgroup 9](#_Toc223444986)

[Table 3.2.1 - Linear Regression Models Adjusted for Age and Age + Creatinine 10](#_Toc223444987)

[Table 3.3 - Biomarker Assessment (Mann–Whitney U Test) on Day 1 and Day 7 – Sepsis Subgroup 11](#_Toc223444988)

[Table 3.3.1 - Linear Regression Models Adjusted for Age and Age + Creatinine 11](#_Toc223444989)

[Table 3.4 - Biomarker Assessment (Mann–Whitney U Test) on Day 1 and Day 7 – mRS Subgroup 12](#_Toc223444990)

[Table 3.4.1 - Linear Regression Models Adjusted for Age and Age + Creatinine 12](#_Toc223444991)

[Table 4.1 - Discriminatory Performance (AUC and ΔAUC) of Model 1 (Age+SOFA) 14](#_Toc223444992)

[Table 4.2 - Discriminatory Performance (AUC and ΔAUC) of Model 2 (Age+GCS) 14](#_Toc223444993)

[Table 4.3 - Discriminatory Performance (AUC and ΔAUC) of Model 3 (APACHE II) 14](#_Toc223444994)

[Table 5.1 - Univariable Logistic Regression Models 16](#_Toc223444995)

[Table 5.2 - Multivariable Logistic Regression Models 16](#_Toc223444996)

# Figure 1 Delta AUC Values


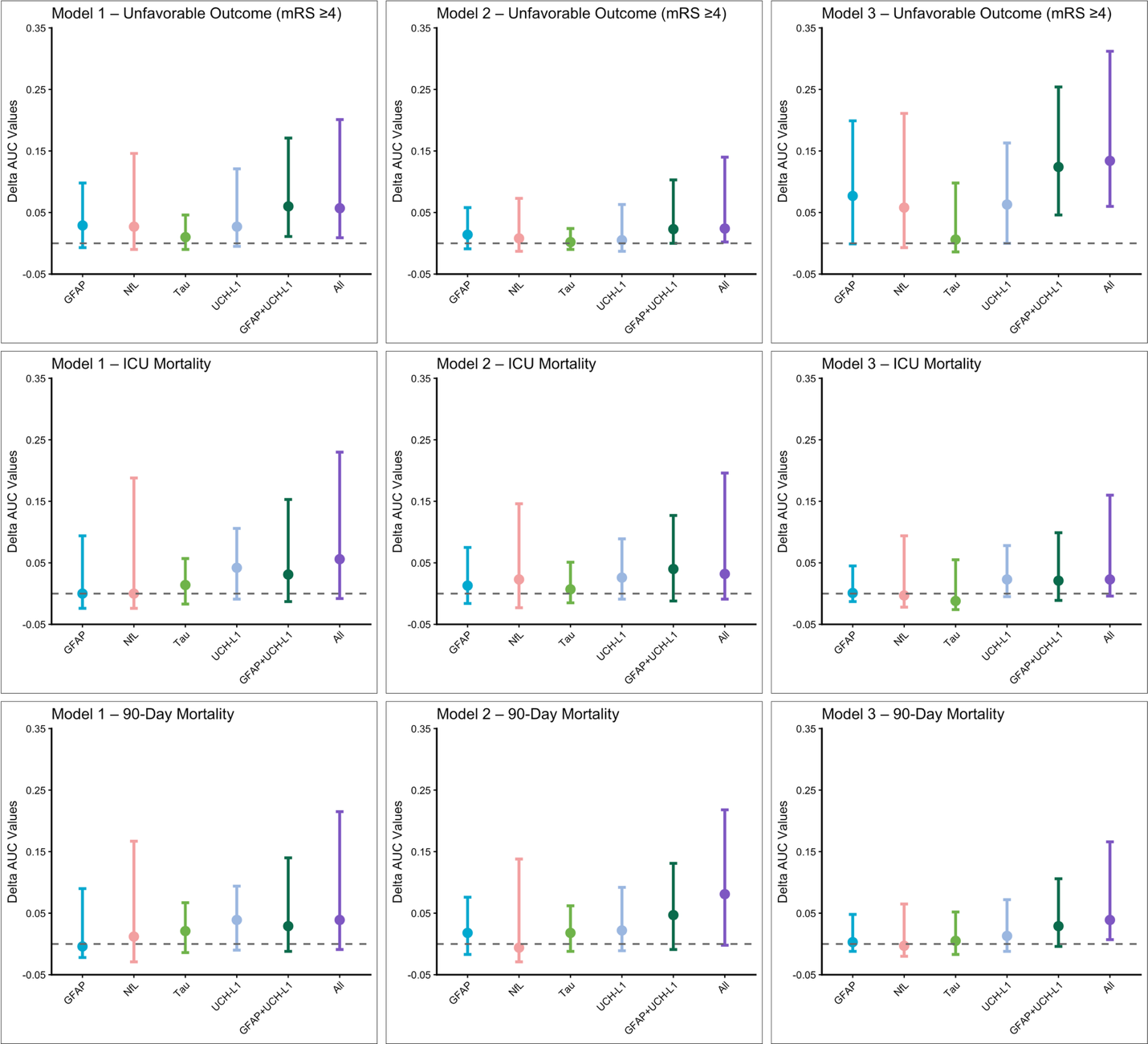


The potential clinical value of brain injury biomarkers is represented by ΔAUC values across prediction models: Model 1 (Age + SOFA), Model 2 (Age + GCS), and Model 3 (APACHE II).

# Table 1.1 - Comorbidities

| **Comorbidities** | **Overall** | |
| --- | --- | --- |
|  | **N** | **%** |
| Myocardial infarction | 16 | 17.8 |
| Heart failure | 48 | 53.3 |
| Peripheral arterial occlusive disease | 8 | 8.9 |
| Cerebrovascular disease | 12 | 35.6 |
| Chronic obstructive pulmonary disease | 16 | 17.8 |
| Peptic ulcer disease | 3 | 3.3 |
| Diabetes mellitus | 27 | 30 |
| Kidney disease | 6 | 6.7 |
| Solid tumor | 13 | 14.4 |
| Liver disease | 4 | 4.4 |

# Table 1.2 - Missing Biomarker Data

| **Biomarker** | **Missing N** | **Total N** | **Missing %** |
| --- | --- | --- | --- |
| GFAP D1 | 29 | 90 | 32.2 |
| NfL D1 | 31 | 90 | 34.4 |
| Tau D1 | 17 | 90 | 18.9 |
| UCH-L1 D1 | 17 | 90 | 18.9 |
| GFAP D7 | 30 | 90 | 33.3 |
| NfL D7 | 46 | 90 | 51.1 |
| Tau D7 | 29 | 90 | 32.2 |
| UCH-L1 D7 | 29 | 90 | 32.2 |

# Table 2.1 - Longitudinal Biomarker Assessment (Wilcoxon Signed-Rank Test); Entire Cohort

| **Biomarker** | **N** | **Median (IQR)** | **Paired N** | **V (signed-rank)** | **z** | **r** | **p-value** |
| --- | --- | --- | --- | --- | --- | --- | --- |
| GFAP (pg/ml) D1 | 61 | 215.1 (136.4–288.4) | 44 | 346 | -1.527 | -0.233 | 0.127 |
| GFAP (pg/ml) D7 | 60 | 227.7 (129.1–516.4) |  |  |  |  |  |
| NFL (pg/ml) D1 | 59 | 224.6 (85.0–467.0) | 36 | 640 | 4.815 | 0.803 | **<0.001** |
| NFL (pg/ml) D7 | 44 | 426.4 (235.7–787.0) |  |  |  |  |  |
| Tau (pg/ml) D1 | 73 | 6.9 (3.7–11.6) | 52 | 337 | -3.051 | -0.427 | **0.002** |
| Tau (pg/ml) D7 | 61 | 6.5 (4.1–11.1) |  |  |  |  |  |
| UCHL1 (pg/ml) D1 | 73 | 49.5 (28.3–98.3) | 52 | 804 | 1.317 | 0.184 | 0.188 |
| UCHL1 (pg/ml) D7 | 61 | 74.8 (46.9–116.2) |  |  |  |  |  |

*Values are median (IQR). Longitudinal changes (D7 vs D1) were assessed using the paired Wilcoxon signed-rank test (two-sided). V is the Wilcoxon signed-rank statistic (sum of positive ranks). z is a normal approximation with tie correction and continuity correction. Effect size r = z/sqrt(n), where n is the number of nonzero paired differences.*

# Table 2.2 - Longitudinal Biomarker Assessment (Wilcoxon Signed-Rank Test); Subgroup Brain Injury

| **Biomarker** | **N (BI Yes)** | **BI Yes Median (IQR)** | **Paired N (BI Yes)** | **V (BI Yes)** | **z (BI Yes)** | **r (BI Yes)** | **p-value (BI Yes)** | **N (BI No)** | **BI No Median (IQR)** | **Paired N (BI No)** | **V (BI No)** | **z (BI No)** | **r (BI No)** | **p-value (BI No)** |
| --- | --- | --- | --- | --- | --- | --- | --- | --- | --- | --- | --- | --- | --- | --- |
| GFAP (pg/ml) D1 | 11 | 404.4 (161.9–852.9) | 11 | 13 | -1.734 | -0.523 | 0.083 | 50 | 208.9 (126.6–268.0) | 33 | 232 | -0.589 | -0.104 | 0.556 |
| GFAP (pg/ml) D7 | 26 | 396.1 (207.4–1510.3) |  |  |  |  |  | 34 | 194.4 (122.7–286.2) |  |  |  |  |  |
| NFL (pg/ml) D1 | 13 | 130.1 (72.1–510.6) | 8 | 36 | 2.45 | 0.866 | **0.014** | 46 | 230.8 (103.0–419.9) | 28 | 384 | 4.11 | 0.777 | **<0.001** |
| NFL (pg/ml) D7 | 14 | 425.4 (165.7–832.8) |  |  |  |  |  | 30 | 426.4 (281.8–783.9) |  |  |  |  |  |
| Tau (pg/ml) D1 | 22 | 6.2 (3.7–11.3) | 18 | 42 | -1.873 | -0.441 | 0.061 | 51 | 7.8 (3.7–13.9) | 34 | 148 | -2.359 | -0.411 | **0.018** |
| Tau (pg/ml) D7 | 27 | 7.3 (4.1–12.0) |  |  |  |  |  | 34 | 5.7 (3.0–11.1) |  |  |  |  |  |
| UCHL1 (pg/ml) D1 | 22 | 89.9 (31.4–116.9) | 18 | 90 | 0.174 | 0.041 | 0.862 | 51 | 48.2 (24.6–90.2) | 34 | 362 | 1.447 | 0.252 | 0.148 |
| UCHL1 (pg/ml) D7 | 27 | 89.6 (53.2–169.4) |  |  |  |  |  | 34 | 70.0 (45.2–102.9) |  |  |  |  |  |

*Values are median (IQR). Within-group longitudinal changes (D7 vs D1) were assessed using the paired Wilcoxon signed-rank test (two-sided), separately for Brain Injury Yes and Brain Injury No. V is the Wilcoxon signed-rank statistic (sum of positive ranks). z is a normal approximation with tie correction and continuity correction. Effect size r = z/sqrt(n), where n is the number of nonzero paired differences.*

# Table 2.2.1 - Analysis of Covariance by Brain Injury Status (Yes/No)

(ANCOVA on log-transformed values adjusting for baseline)

| Biomarker | Adjusted Geometric Mean (Yes) | Adjusted Geometric Mean (No) | Ratio (No vs Yes) (95% CI) | p-Value |
| --- | --- | --- | --- | --- |
| GFAP (D1-D7) | 155 (109–219) | 228 (188–276) | 1.47 (0.98–2.10) | 0.06 |
| NFL (D1-D7) | 388.6 (288.8–522.3 | 398.9 (340.4–468.6 | 1.03 (0.65–1.62) | 0.871 |
| Tau (D1-D7) | 5.83 (4.64–7.33 | 6.01 (5.09–7.10) | 1.03 (0.69–1.53) | 0.832 |
| UCHL1 (D1-D7) | 59.2 (46.8–74.7) | 66.6 (56.2–78.9) | 1.13 (0.75–1.69) | 0.413 |

Analysis of covariance was performed for each biomarker using log-transformed Day 7 levels as dependent variables and log-transformed baseline (Day 1) values. Values are presented as adjusted geometric means (GM) with 95% confidence intervals. Ratios represent exponentiated adjusted group differences.

# Table 2.3 - Longitudinal Biomarker Assessment (Wilcoxon Signed-Rank Test); Subgroup Delirium

| **Biomarker** | **N** | **Delirium Yes Median (IQR)** | **Paired N (Yes)** | **V (Yes)** | **z (Yes)** | **r (Yes)** | **p-value (Yes)** | **N** | **Delirium No Median (IQR)** | **Paired N (No)** | **V (No)** | **z (No)** | **r (No)** | **p-value (No)** |
| --- | --- | --- | --- | --- | --- | --- | --- | --- | --- | --- | --- | --- | --- | --- |
| GFAP (pg/ml) D1 | 37 | 234.9 (136.5–367.1) | 27 | 121 | -1.622 | -0.312 | 0.105 | 24 | 185.5 (131.5–267.2) | 17 | 64 | -0.181 | -0.045 | 0.856 |
| GFAP (pg/ml) D7 | 34 | 212.5 (114.4–441.9) |  |  |  |  |  | 26 | 241.3 (157.4–605.8) |  |  |  |  |  |
| *Brain Injury excluded | 33 | 229.4 (136.5–282.7) | 23 | 113 | -0.745 | -0.155 | 0.456 | 17 | 164.6 (126.6–253.0) | 10 | 25 | 0.237 | 0.079 | 0.813 |
| *Brain Injury excluded | 24 | 191.7 (110.6–364.0) |  |  |  |  |  | 10 | 209.4 (126.8–254.8) |  |  |  |  |  |
| NFL (pg/ml) D1 | 36 | 255.5 (109.8–589.7) | 23 | 259 | 3.665 | 0.764 | **<0.001** | 23 | 132.9 (80.6–337.8) | 13 | 90 | 3.075 | 0.853 | **0.002** |
| NFL (pg/ml) D7 | 27 | 552.5 (281.8–925.9) |  |  |  |  |  | 17 | 335.0 (178.7–657.8) |  |  |  |  |  |
| *Brain Injury excluded | 31 | 260.2 (143.6–574.2) | 20 | 194 | 3.304 | 0.739 | **<0.001** | 15 | 132.9 (85.0–208.0) | 8 | 35 | 2.31 | 0.817 | **0.021** |
| *Brain Injury excluded | 22 | 504.7 (281.8–922.1) |  |  |  |  |  | 8 | 338.7 (255.6–572.5) |  |  |  |  |  |
| Tau (pg/ml) D1 | 43 | 8.7 (3.7–16.4) | 31 | 69 | -3.498 | -0.628 | **<0.001** | 30 | 6.2 (3.5–10.5) | 21 | 98 | -0.243 | -0.054 | 0.808 |
| Tau (pg/ml) D7 | 35 | 5.7 (2.8–12.0) |  |  |  |  |  | 26 | 7.5 (4.3–9.3) |  |  |  |  |  |
| *Brain Injury excluded | 34 | 7.9 (3.9–15.7) | 24 | 43 | -3.043 | -0.621 | **0.002** | 17 | 7.8 (3.5–9.7) | 10 | 27 | 0.474 | 0.158 | 0.636 |
| *Brain Injury excluded | 24 | 5.1 (2.7–9.8) |  |  |  |  |  | 10 | 8.0 (4.3–14.4) |  |  |  |  |  |
| UCHL1 (pg/ml) D1 | 43 | 48.3 (25.6–109.2) | 31 | 308 | 1.166 | 0.209 | 0.244 | 30 | 56.2 (28.3–98.3) | 21 | 122 | 0.616 | 0.138 | 0.538 |
| UCHL1 (pg/ml) D7 | 35 | 84.1 (56.0–123.0) |  |  |  |  |  | 26 | 64.7 (45.2–116.2) |  |  |  |  |  |
| *Brain Injury excluded | 34 | 47.7 (25.6–90.2) | 24 | 214 | 1.814 | 0.37 | 0.070 | 17 | 49.5 (24.6–84.4) | 10 | 21 | -0.118 | -0.039 | 0.906 |
| *Brain Injury excluded | 24 | 79.2 (49.8–107.6) |  |  |  |  |  | 10 | 54.8 (45.2–83.1) |  |  |  |  |  |

*Values are median (IQR). Within-group longitudinal changes (D7 vs D1) were assessed using the paired Wilcoxon signed-rank test (two-sided), separately for Delirium Yes and Delirium No and repeated after excluding patients with Brain Injury. V is the Wilcoxon signed-rank statistic (sum of positive ranks). z is a normal approximation with tie correction and continuity correction. Effect size r = z/sqrt(n), where n is the number of nonzero paired differences.*

# Table 2.3.1 - Analysis of Covariance by Delirium Status (Yes/No)

(ANCOVA on log-transformed values adjusting for baseline)

| Biomarker | Adjusted Geometric Mean (Yes) | Adjusted Geometric Mean (No) | Ratio (No vs Yes) (95% CI) | p-Value |
| --- | --- | --- | --- | --- |
| GFAP (D1-D7) | 197.5 (159.3–244.9) | 221.8 (169.0–291.5) | 1.12 (0.83–1.50) | 0.503 |
| NFL (D1-D7) | 398.9 (333.3–472.8) | 396.1 (312.5–499.7) | 0.99 (0.69–1.42) | 0.964 |
| Tau (D1-D7) | 5.17 (4.39–6.09) | 7.31 (5.99–8.92) | 1.41 (1.09–1.83) | **0.009** |
| UCHL1 (D1-D7) | 67.8 (56.6–80.9) | 58.7 (47.3–72.8) | 0.87 (0.63–1.19) | 0.314 |

Analysis of covariance was performed for each biomarker using log-transformed Day 7 levels as dependent variables and log-transformed baseline (Day 1) values. Values are presented as adjusted geometric means (GM) with 95% confidence intervals. Ratios represent exponentiated adjusted group differences.

# Table 2.4 - Longitudinal Biomarker Assessment (Wilcoxon Signed-Rank Test); Subgroup Sepsis

| **Biomarker** | **N** | **Sepsis Yes Median (IQR)** | **Paired N (Sepsis Y)** | **V (Sepsis Y)** | **z (Sepsis Y)** | **r (Sepsis Y)** | **p-value (Sepsis Y)** | **N** | **Sepsis No Median (IQR)** | **Paired N (Sepsis N)** | **V (Sepsis N)** | **z (Sepsis N)** | **r (Sepsis N)** | **p-value (N)** |
| --- | --- | --- | --- | --- | --- | --- | --- | --- | --- | --- | --- | --- | --- | --- |
| GFAP (pg/ml) D1 | 42 | 222.3 (137.8–274.7) | 33 | 229 | -0.645 | -0.114 | 0.519 | 19 | 206.1 (126.6–567.2) | 11 | 13 | -1.734 | -0.523 | 0.083 |
| GFAP (pg/ml) D7 | 44 | 211.7 (124.8–427.9) |  |  |  |  |  | 16 | 291.3 (202.3–804.6) |  |  |  |  |  |
| *Brain Injury excludedD1 | 38 | 235.1 (137.8–274.7) | 29 | 173 | -0.672 | -0.127 | 0.502 | 12 | 155.2 (122.6–220.5) | 4 | 4 | -0.183 | -0.091 | 0.855 |
| *Brain Injury excludedD7 | 30 | 194.4 (122.7–294.0) |  |  |  |  |  | 4 | 177.2 (116.8–245.9) |  |  |  |  |  |
| NFL (pg/ml) D1 | 40 | 221.5 (82.8–485.7) | 27 | 361 | 4.12 | 0.793 | **<0.001** | 19 | 237.1 (111.4–419.9) | 9 | 44 | 2.488 | 0.829 | **0.013** |
| NFL (pg/ml) D7 | 33 | 433.8 (281.8–783.9) |  |  |  |  |  | 11 | 361.1 (124.3–832.8) |  |  |  |  |  |
| *Brain Injury excludedD1 | 34 | 237.7 (88.2–480.5) | 24 | 285 | 3.843 | 0.784 | **<0.001** | 12 | 196.2 (111.8–254.5) | 4 | 9 | 1.278 | 0.639 | 0.201 |
| *Brain Injury excludedD7 | 26 | 504.7 (335.0–790.0) |  |  |  |  |  | 4 | 177.9 (123.2–296.3) |  |  |  |  |  |
| Tau (pg/ml) D1 | 48 | 8.3 (3.8–14.0) | 38 | 167 | -2.776 | -0.456 | **0.006** | 25 | 6.2 (3.7–11.1) | 14 | 32 | -1.256 | -0.336 | 0.209 |
| Tau (pg/ml) D7 | 45 | 7.7 (4.1–12.0) |  |  |  |  |  | 16 | 5.7 (4.0–8.9) |  |  |  |  |  |
| *Brain Injury excludedD1 | 39 | 8.9 (3.9–14.1) | 30 | 127 | -1.946 | -0.361 | 0.052 | 12 | 6.1 (3.2–10.5) | 4 | 1 | -1.278 | -0.639 | 0.201 |
| *Brain Injury excludedD7 | 30 | 7.5 (3.6–11.8) |  |  |  |  |  | 4 | 4.1 (2.1–5.7) |  |  |  |  |  |
| UCHL1 (pg/ml) D1 | 48 | 52.2 (29.7–106.1) | 38 | 405 | 0.8 | 0.131 | 0.424 | 25 | 46.8 (21.4–84.4) | 14 | 71 | 1.13 | 0.302 | 0.258 |
| UCHL1 (pg/ml) D7 | 45 | 83.1 (46.9–123.0) |  |  |  |  |  | 16 | 64.2 (39.0–102.6) |  |  |  |  |  |
| *Brain Injury excludedD1 | 39 | 49.5 (31.0–95.9) | 30 | 281 | 1.362 | 0.253 | 0.173 | 12 | 25.8 (8.7–62.5) | 4 | 6 | 0.183 | 0.091 | 0.855 |
| *Brain Injury excludedD7 | 30 | 78.4 (46.9–112.3) |  |  |  |  |  | 4 | 35.2 (22.9–66.2) |  |  |  |  |  |

*Values are median (IQR). Within-group longitudinal changes (D7 vs D1) were assessed using the paired Wilcoxon signed-rank test (two-sided), separately for Sepsis Yes and Sepsis No, in the full cohort and after excluding patients with Brain Injury. V is the Wilcoxon signed-rank statistic (sum of positive ranks). z is a normal approximation with tie correction and continuity correction. Effect size r = z/sqrt(n), where n is the number of nonzero paired differences.*

# Table 2.4.1 - Analysis of Covariance by Sepsis Status (Yes/No)

| Biomarker | Adjusted Geometric Mean (Yes) | Adjusted Geometric Mean (No) | Ratio (No vs Yes) (95% CI) | p-Value |
| --- | --- | --- | --- | --- |
| GFAP (D1-D7) | 200.4 (166–241) | 197.9 (120–324) | 1.01 (0.51–1.99) | 0.965 |
| NFL (D1-D7) | 445.6 (376–531) | 270.5 (175–417) | 1.65 (1.05–2.59) | **0.038** |
| Tau (D1-D7) | 6.41 (5.27–7.81) | 4.49 (2.61–7.73) | 1.43 (0.68–2.99) | 0.219 |
| UCHL1 (D1-D7) | 67.6 (55.3–82.3) | 64.8 (36.3–115.6) | 1.04 (0.48–2.27) | 0.896 |

**Patients with Brain Injury excluded*

Analysis of covariance was performed for each biomarker using log-transformed Day 7 levels as dependent variables and log-transformed baseline (Day 1) values. Values are presented as adjusted geometric means (GM) with 95% confidence intervals. Ratios represent exponentiated adjusted group differences (No vs. Yes XXX).

# Table 2.5 - Longitudinal Biomarker Assessment (Wilcoxon Signed-Rank Test); Subgroup mRS

| **Biomarker** | **N (mRS ≥4)** | **mRS ≥4 Median (IQR)** | **Paired N (mRS ≥4)** | **V (mRS ≥4)** | **z (mRS ≥4)** | **r (mRS ≥4)** | **p-value (mRS ≥4)** | **N (mRS ≤3)** | **mRS ≤3 Median (IQR)** | **Paired N (mRS ≤3)** | **V (mRS ≤3)** | **z (mRS ≤3)** | **r (mRS ≤3)** | **p-value (mRS ≤3)** |
| --- | --- | --- | --- | --- | --- | --- | --- | --- | --- | --- | --- | --- | --- | --- |
| GFAP (pg/ml) D1 | 44 | 255.3 (162.5–407.4) | 36 | 210 | -1.712 | -0.289 | 0.087 | 17 | 136.5 (82.5–206.1) | 8 | 20 | 0.21 | 0.074 | 0.834 |
| GFAP (pg/ml) D7 | 50 | 256.9 (157.4–605.8) |  |  |  |  |  | 10 | 110.9 (89.9–227.7) |  |  |  |  |  |
| *Brain Injury excluded GFAP (pg/ml) D1 | 35 | 253.0 (150.2–317.2) | 27 | 127 | -1.219 | -0.239 | 0.223 | 15 | 136.5 (77.4–206.1) | 6 | 19 | 1.677 | 0.685 | 0.093 |
| *Brain Injury excluded GFAP (pg/ml) D7 | 28 | 197.3 (129.1–299.1) |  |  |  |  |  | 6 | 110.9 (97.1–227.7) |  |  |  |  |  |
| NFL (pg/ml) D1 | 42 | 332.4 (132.9–510.6) | 28 | 400 | 4.475 | 0.846 | **<0.001** | 17 | 85.0 (52.1–147.0) | 8 | 29 | 1.47 | 0.52 | 0.141 |
| NFL (pg/ml) D7 | 35 | 552.5 (332.5–922.1) |  |  |  |  |  | 9 | 178.7 (146.0–298.4) |  |  |  |  |  |
| *Brain Injury excluded NFL (pg/ml) D1 | 31 | 326.9 (186.0–480.5) | 22 | 247 | 3.896 | 0.831 | **<0.001** | 15 | 111.4 (52.1–155.3) | 6 | 15 | 0.839 | 0.342 | 0.402 |
| *Brain Injury excluded NFL (pg/ml) D7 | 24 | 504.7 (351.7–856.0) |  |  |  |  |  | 6 | 171.0 (146.0–231.5) |  |  |  |  |  |
| Tau (pg/ml) D1 | 53 | 8.9 (4.8–14.1) | 43 | 223 | -2.851 | -0.44 | **0.004** | 20 | 4.8 (2.8–8.7) | 9 | 14 | -0.948 | -0.316 | 0.343 |
| Tau (pg/ml) D7 | 51 | 7.7 (4.3–14.4) |  |  |  |  |  | 10 | 3.3 (2.1–4.7) |  |  |  |  |  |
| *Brain Injury excluded Tau (pg/ml) D1 | 36 | 9.3 (4.9–15.1) | 28 | 107 | -1.958 | -0.377 | 0.051 | 15 | 5.3 (2.8–9.5) | 6 | 3 | -1.468 | -0.599 | 0.142 |
| *Brain Injury excluded Tau (pg/ml) D7 | 28 | 7.5 (4.3–13.1) |  |  |  |  |  | 6 | 2.7 (1.6–4.3) |  |  |  |  |  |
| UCHL1 (pg/ml) D1 | 53 | 70.9 (41.4–111.3) | 43 | 513 | 0.763 | 0.118 | 0.446 | 20 | 23.9 (6.4–39.1) | 9 | 37 | 1.659 | 0.553 | 0.097 |
| UCHL1 (pg/ml) D7 | 51 | 84.1 (61.4–151.1) |  |  |  |  |  | 10 | 22.9 (18.7–53.2) |  |  |  |  |  |
| *Brain Injury excluded UCHL1 (pg/ml) D1 | 36 | 55.3 (35.7–96.8) | 28 | 237 | 1.141 | 0.22 | 0.254 | 15 | 22.3 (6.5–36.3) | 6 | 15 | 0.839 | 0.342 | 0.402 |
| *Brain Injury excluded UCHL1 (pg/ml) D7 | 28 | 82.8 (58.7–107.6) |  |  |  |  |  | 6 | 22.9 (21.4–40.3) |  |  |  |  |  |

*Values are median (IQR). Within-group longitudinal changes (D7 vs D1) were assessed using the paired Wilcoxon signed-rank test (two-sided), in the total cohort and repeated after excluding patients with Brain Injury. V is the Wilcoxon signed-rank statistic (sum of positive ranks). z is a normal approximation with tie correction and continuity correction. Effect size r = z/sqrt(n), where n is the number of nonzero paired differences.*

# Table 2.5.1 - Analysis of Covariance by mRS Status (mRS ≥4/ mRS ≤3)

| Biomarker | Adjusted Geometric Mean (Yes) | Adjusted Geometric Mean (No) | Ratio (No vs Yes) (95% CI) | p-Value |
| --- | --- | --- | --- | --- |
| GFAP (D1-D7) | 167.0 (110.2–252.9) | 216.8 (179.4–261.7) | 1.30 (0.82–2.05) | 0.266 |
| NFL (D1-D7) | 304.8 (226.6–409.9) | 427.9 (368.0–497.3) | 1.40 (0.99–1.98) | 0.055 |
| Tau (D1-D7) | 4.71 (3.35–6.61) | 6.24 (5.39–7.23) | 1.33 (0.90–1.96) | 0.139 |
| UCHL1 (D1-D7) | 53.3 (36.3–78.3) | 66.6 (56.8–78.1) | 1.25 (0.80–1.94) | 0.307 |

Analysis of covariance was performed for each biomarker using log-transformed Day 7 levels as dependent variables and log-transformed baseline (Day 1) values. Values are presented as adjusted geometric means (GM) with 95% confidence intervals. Ratios represent exponentiated adjusted group differences (No vs. Yes XXX).

# Table 2.5.2 - Analysis of Covariance by mRS Status (mRS ≥4/ mRS ≤3), Brain Injury excluded

| Biomarker | Adjusted Geometric Mean (Yes) | Adjusted Geometric Mean (No) | Ratio (No vs Yes) (95% CI) | p-Value |
| --- | --- | --- | --- | --- |
| GFAP (D1-D7) | 191.3 (158.1–231.4) | 242.5 (157.1–374.3) | 0.79 (0.49–1.26) | 0.329 |
| NFL (D1-D7) | 463.1 (389.2–550.6) | 279.2 (198.7–392.3) | 1.66 (1.15–2.40) | **0.013** |
| Tau (D1-D7) | 6.61 (5.40–8.08) | 4.39 (2.78–6.94) | 1.50 (0.92–2.45) | 0.113 |
| UCHL1 (D1-D7) | 71.16 (58.09–87.18) | 51.01 (31.72–82.02) | 1.40 (0.84–2.33) | 0.212 |

**Patients with Brain Injury excluded*

Analysis of covariance was performed for each biomarker using log-transformed Day 7 levels as dependent variables and log-transformed baseline (Day 1) values. Values are presented as adjusted geometric means (GM) with 95% confidence intervals. Ratios represent exponentiated adjusted group differences.

# Table 3.1 - Biomarker Assessment (Mann–Whitney U Test) on Day 1 and Day 7 – Brain Injury Subgroup

| **Biomarker** | **N** | **Brain Injury median (IQR)** | **Mean rank (Brain Injury)** | **N** | **No Brain Injury median (IQR)** | **Mean rank (No Brain Injury)** | **U** | **z** | **r** | **p-value** |
| --- | --- | --- | --- | --- | --- | --- | --- | --- | --- | --- |
| GFAP (pg/ml) D1 | 11 | 404.4 (161.9–852.9) | 40.73 | 50 | 208.9 (126.6–268.0) | 28.86 | 382 | 1.998 | 0.256 | **0.046** |
| GFAP (pg/ml) D7 | 26 | 396.1 (207.4–1510.3) | 36.96 | 34 | 194.4 (122.7–286.2) | 25.56 | 610 | 2.499 | 0.323 | **0.012** |
| NFL (pg/ml) D1 | 13 | 130.1 (72.1–510.6) | 29.38 | 46 | 230.8 (103.0–419.9) | 30.17 | 291 | -0.137 | -0.018 | 0.891 |
| NFL (pg/ml) D7 | 14 | 425.4 (165.7–832.8) | 21.36 | 30 | 426.4 (281.8–783.9) | 23.03 | 194 | -0.391 | -0.059 | 0.696 |
| Tau (pg/ml) D1 | 22 | 6.2 (3.7–11.3) | 35.64 | 51 | 7.8 (3.7–13.9) | 37.59 | 531 | -0.355 | -0.042 | 0.723 |
| Tau (pg/ml) D7 | 27 | 7.3 (4.1–12.0) | 33.11 | 34 | 5.7 (3.0–11.1) | 29.32 | 516 | 0.82 | 0.105 | 0.412 |
| UCHL1 (pg/ml) D1 | 22 | 89.9 (31.4–116.9) | 43.64 | 51 | 48.2 (24.6–90.2) | 34.14 | 707 | 1.749 | 0.205 | 0.080 |
| UCHL1 (pg/ml) D7 | 27 | 89.6 (53.2–169.4) | 33.85 | 34 | 70.0 (45.2–102.9) | 28.74 | 536 | 1.111 | 0.142 | 0.267 |

*Values are median (IQR). Mean ranks, Mann–Whitney U, z (normal approximation with tie correction and continuity correction), and effect size r = z/sqrt(N) are reported. Two-sided p-values are derived from z.*

# Table 3.1.1 - Linear Regression Models Adjusted for Age and Age + Creatinine

| **Biomarker** | **N (age)** | **Beta (CI) age** | **p-value age** | **Fold Change age** | **Percent Change age** | **N (age+crea)** | **Beta (CI) age+crea** | **p-value age+crea** | **Fold Change age+crea** | **Percent Change age+crea** |
| --- | --- | --- | --- | --- | --- | --- | --- | --- | --- | --- |
| GFAP (pg/ml) D1 | 61 | 1.021 (0.483, 1.560) | **0.000** | 2.777 | 177.693 | 61 | 1.050 (0.500, 1.600) | **0.000** | 2.857 | 185.696 |
| GFAP (pg/ml) D7 | 60 | 0.902 (0.387, 1.416) | **0.001** | 2.464 | 146.352 | 60 | 0.811 (0.283, 1.338) | **0.003** | 2.249 | 124.917 |
| NFL (pg/ml) D1 | 59 | 0.393 (-0.227, 1.014) | 0.210 | 1.482 | 48.166 | 59 | 0.541 (-0.062, 1.144) | 0.078 | 1.718 | 71.811 |
| NFL (pg/ml) D7 | 44 | 0.238 (-0.263, 0.739) | 0.343 | 1.268 | 26.839 | 44 | 0.345 (-0.149, 0.839) | 0.166 | 1.412 | 41.183 |
| Tau (pg/ml) D1 | 73 | 0.133 (-0.326, 0.592) | 0.566 | 1.142 | 14.212 | 73 | 0.273 (-0.152, 0.699) | 0.205 | 1.314 | 31.413 |
| Tau (pg/ml) D7 | 61 | 0.406 (-0.039, 0.851) | 0.073 | 1.501 | 50.131 | 61 | 0.498 (0.050, 0.946) | **0.030** | 1.645 | 64.501 |
| UCHL1 (pg/ml) D1 | 73 | 0.751 (0.205, 1.297) | **0.008** | 2.119 | 111.923 | 73 | 0.896 (0.376, 1.416) | **0.001** | 2.450 | 144.998 |
| UCHL1 (pg/ml) D7 | 61 | 0.313 (-0.120, 0.747) | 0.154 | 1.368 | 36.815 | 61 | 0.423 (-0.008, 0.853) | 0.054 | 1.526 | 52.585 |

*Biomarkers were log-transformed and analyzed using multivariable linear regression adjusted for age and age plus creatinine. β coefficients (95% CI) are shown; fold and percent changes were derived from exponentiated coefficients.*

# Table 3.2 - Biomarker Assessment (Mann–Whitney U Test) on Day 1 and Day 7 – Delirium Subgroup

| **Biomarker** | **N** | **Delirium median (IQR)** | **Mean rank (Delirium)** | **N** | **No Delirium median (IQR)** | **Mean rank (No Delirium)** | **U** | **z** | **r** | **p-value** |
| --- | --- | --- | --- | --- | --- | --- | --- | --- | --- | --- |
| GFAP (pg/ml) D1 | 37 | 234.9 (136.5–367.1) | 32.57 | 24 | 185.5 (131.5–267.2) | 28.58 | 502 | 0.849 | 0.109 | 0.396 |
| *Brain Injury excluded | 33 | 229.4 (136.5–282.7) | 27.7 | 17 | 164.6 (126.6–253.0) | 21.24 | 353 | 1.475 | 0.209 | 0.140 |
| *Sepsis Subgroup | 27 | 255.7 (150.2–367.1) | 24.11 | 15 | 164.6 (121.8–253.0) | 16.8 | 273 | 1.837 | 0.284 | 0.066 |
| GFAP (pg/ml) D7 | 34 | 212.5 (114.4–441.9) | 28.24 | 26 | 241.3 (157.4–605.8) | 33.46 | 365 | -1.141 | -0.147 | 0.254 |
| *Brain Injury excluded | 24 | 191.7 (110.6–364.0) | 17.33 | 10 | 209.4 (126.8–254.8) | 17.9 | 116 | -0.132 | -0.023 | 0.895 |
| *Sepsis Subgroup | 28 | 222.4 (120.6–546.3) | 22.89 | 16 | 211.7 (129.8–274.4) | 21.81 | 235 | 0.256 | 0.039 | 0.798 |
| NFL (pg/ml) D1 | 36 | 255.5 (109.8–589.7) | 32.92 | 23 | 132.9 (80.6–337.8) | 25.43 | 519 | 1.624 | 0.211 | 0.104 |
| *Brain Injury excluded | 31 | 260.2 (143.6–574.2) | 26.68 | 15 | 132.9 (85.0–208.0) | 16.93 | 331 | 2.296 | 0.339 | **0.022** |
| *Sepsis Subgroup | 27 | 368.2 (107.3–668.9) | 23.15 | 13 | 103.0 (80.6–186.0) | 15 | 247 | 2.05 | 0.324 | **0.040** |
| NFL (pg/ml) D7 | 27 | 552.5 (281.8–925.9) | 24.67 | 17 | 335.0 (178.7–657.8) | 19.06 | 288 | 1.398 | 0.211 | 0.162 |
| *Brain Injury excluded | 22 | 504.7 (281.8–922.1) | 16.86 | 8 | 338.7 (255.6–572.5) | 11.75 | 118 | 1.383 | 0.253 | 0.167 |
| *Sepsis Subgroup | 22 | 644.1 (366.0–925.9) | 19.91 | 11 | 332.5 (165.7–433.8) | 11.18 | 185 | 2.425 | 0.422 | **0.015** |
| Tau (pg/ml) D1 | 43 | 8.7 (3.7–16.4) | 39.33 | 30 | 6.2 (3.5–10.5) | 33.67 | 745 | 1.116 | 0.131 | 0.265 |
| *Brain Injury excluded | 34 | 7.9 (3.9–15.7) | 27.41 | 17 | 7.8 (3.5–9.7) | 23.18 | 337 | 0.949 | 0.133 | 0.343 |
| *Sepsis Subgroup | 32 | 9.3 (3.8–16.2) | 26.03 | 16 | 5.9 (4.2–10.4) | 21.44 | 305 | 1.061 | 0.153 | 0.289 |
| Tau (pg/ml) D7 | 35 | 5.7 (2.8–12.0) | 29.74 | 26 | 7.5 (4.3–9.3) | 32.69 | 411 | -0.634 | -0.081 | 0.526 |
| *Brain Injury excluded | 24 | 5.1 (2.7–9.8) | 15.83 | 10 | 8.0 (4.3–14.4) | 21.5 | 80 | -1.493 | -0.256 | 0.135 |
| *Sepsis Subgroup | 29 | 7.3 (3.0–12.0) | 22.28 | 16 | 7.8 (4.2–13.1) | 24.31 | 211 | -0.486 | -0.072 | 0.627 |
| UCHL1 (pg/ml) D1 | 43 | 48.3 (25.6–109.2) | 36.91 | 30 | 56.2 (28.3–98.3) | 37.13 | 641 | -0.039 | -0.005 | 0.969 |
| *Brain Injury excluded | 34 | 47.7 (25.6–90.2) | 26.03 | 17 | 49.5 (24.6–84.4) | 25.94 | 290 | 0.01 | 0.001 | 0.992 |
| *Sepsis Subgroup | 32 | 52.2 (31.3–116.8) | 25.78 | 16 | 52.6 (25.4–96.6) | 21.94 | 297 | 0.886 | 0.128 | 0.376 |
| UCHL1 (pg/ml) D7 | 35 | 84.1 (56.0–123.0) | 32.03 | 26 | 64.7 (45.2–116.2) | 29.62 | 491 | 0.518 | 0.066 | 0.605 |
| *Brain Injury excluded | 24 | 79.2 (49.8–107.6) | 18.5 | 10 | 54.8 (45.2–83.1) | 15.1 | 144 | 0.888 | 0.152 | 0.374 |
| *Sepsis Subgroup | 29 | 96.4 (63.4–123.0) | 24.03 | 16 | 72.6 (26.9–134.1) | 21.12 | 262 | 0.699 | 0.104 | 0.484 |

*Values are median (IQR). Mean ranks, Mann–Whitney U, z (normal approximation with tie correction and continuity correction), and effect size r = z/sqrt(N) are reported. Two-sided p-values are derived from z.*

# Table 3.2.1 - Linear Regression Models Adjusted for Age and Age + Creatinine

| **Biomarker** | **N (age)** | **Beta (CI) age** | **p-value age** | **Fold Change age** | **Percent Change age** | **N (age+crea)** | **Beta (CI) age+crea** | **p-value age+crea** | **Fold Change age+crea** | **Percent Change age+crea** |
| --- | --- | --- | --- | --- | --- | --- | --- | --- | --- | --- |
| GFAP (pg/ml) D1 | 61 | 0.160 (-0.283, 0.603) | 0.472 | 1.174 | 17.396 | 61 | 0.173 (-0.288, 0.633) | 0.457 | 1.188 | 18.830 |
| *Brain Injury excluded | 50 | 0.352 (-0.047, 0.751) | 0.082 | 1.422 | 42.226 | 50 | 0.318 (-0.092, 0.728) | 0.125 | 1.374 | 37.435 |
| *Sepsis Subgroup | 42 | 0.418 (-0.036, 0.871) | 0.070 | 1.519 | 51.883 | 42 | 0.401 (-0.067, 0.869) | 0.091 | 1.494 | 49.374 |
| GFAP (pg/ml) D7 | 60 | -0.367 (-0.889, 0.156) | 0.166 | 0.693 | -30.690 | 60 | -0.244 (-0.776, 0.289) | 0.363 | 0.784 | -21.625 |
| *Brain Injury excluded | 34 | 0.113 (-0.460, 0.686) | 0.691 | 1.119 | 11.935 | 34 | 0.138 (-0.440, 0.716) | 0.629 | 1.148 | 14.811 |
| *Sepsis Subgroup | 44 | 0.082 (-0.545, 0.709) | 0.792 | 1.086 | 8.576 | 44 | 0.135 (-0.491, 0.761) | 0.665 | 1.145 | 14.450 |
| NFL (pg/ml) D1 | 59 | 0.276 (-0.211, 0.764) | 0.261 | 1.318 | 31.823 | 59 | 0.126 (-0.375, 0.627) | 0.617 | 1.134 | 13.380 |
| *Brain Injury excluded | 46 | 0.527 (-0.011, 1.065) | 0.055 | 1.694 | 69.386 | 46 | 0.346 (-0.194, 0.885) | 0.203 | 1.413 | 41.287 |
| *Sepsis Subgroup | 40 | 0.644 (0.028, 1.260) | **0.041** | 1.904 | 90.383 | 40 | 0.452 (-0.155, 1.059) | 0.140 | 1.571 | 57.138 |
| NFL (pg/ml) D7 | 44 | 0.178 (-0.256, 0.613) | 0.412 | 1.195 | 19.531 | 44 | 0.079 (-0.366, 0.525) | 0.721 | 1.083 | 8.269 |
| *Brain Injury excluded | 30 | 0.271 (-0.289, 0.831) | 0.329 | 1.312 | 31.158 | 30 | 0.154 (-0.362, 0.670) | 0.544 | 1.167 | 16.697 |
| *Sepsis Subgroup | 33 | 0.485 (0.029, 0.942) | **0.038** | 1.625 | 62.463 | 33 | 0.391 (-0.063, 0.845) | 0.088 | 1.479 | 47.882 |
| Tau (pg/ml) D1 | 73 | 0.203 (-0.203, 0.608) | 0.322 | 1.225 | 22.460 | 73 | -0.007 (-0.402, 0.387) | 0.970 | 0.993 | -0.744 |
| *Brain Injury excluded | 51 | 0.204 (-0.279, 0.688) | 0.400 | 1.227 | 22.660 | 51 | 0.046 (-0.404, 0.496) | 0.838 | 1.047 | 4.699 |
| *Sepsis Subgroup | 48 | 0.251 (-0.277, 0.779) | 0.343 | 1.285 | 28.516 | 48 | 0.094 (-0.407, 0.595) | 0.707 | 1.099 | 9.866 |
| Tau (pg/ml) D7 | 61 | -0.179 (-0.612, 0.254) | 0.412 | 0.836 | -16.383 | 61 | -0.275 (-0.719, 0.169) | 0.221 | 0.760 | -24.008 |
| *Brain Injury excluded | 34 | -0.487 (-1.040, 0.066) | 0.082 | 0.615 | -38.547 | 34 | -0.549 (-1.059, -0.039) | **0.036** | 0.577 | -42.261 |
| *Sepsis Subgroup | 45 | -0.193 (-0.752, 0.365) | 0.488 | 0.824 | -17.589 | 45 | -0.224 (-0.789, 0.342) | 0.429 | 0.800 | -20.043 |
| UCHL1 (pg/ml) D1 | 73 | -0.078 (-0.587, 0.431) | 0.760 | 0.925 | -7.528 | 73 | -0.294 (-0.804, 0.217) | 0.255 | 0.746 | -25.449 |
| *Brain Injury excluded | 51 | 0.158 (-0.477, 0.792) | 0.620 | 1.171 | 17.068 | 51 | -0.036 (-0.635, 0.563) | 0.904 | 0.965 | -3.534 |
| *Sepsis Subgroup | 48 | 0.273 (-0.316, 0.862) | 0.356 | 1.314 | 31.370 | 48 | 0.095 (-0.462, 0.652) | 0.733 | 1.100 | 9.964 |
| UCHL1 (pg/ml) D7 | 61 | 0.014 (-0.407, 0.434) | 0.948 | 1.014 | 1.375 | 61 | -0.093 (-0.521, 0.334) | 0.664 | 0.911 | -8.914 |
| *Brain Injury excluded | 34 | 0.147 (-0.409, 0.704) | 0.593 | 1.159 | 15.880 | 34 | 0.089 (-0.432, 0.611) | 0.729 | 1.094 | 9.355 |
| *Sepsis Subgroup | 45 | 0.174 (-0.358, 0.706) | 0.512 | 1.190 | 19.039 | 45 | 0.127 (-0.404, 0.658) | 0.632 | 1.135 | 13.513 |

*Biomarkers were log-transformed and analyzed using multivariable linear regression adjusted for age and age plus creatinine. β coefficients (95% CI) are shown; fold and percent changes were derived from exponentiated coefficients.*

# Table 3.3 - Biomarker Assessment (Mann–Whitney U Test) on Day 1 and Day 7 – Sepsis Subgroup

| **Biomarker** | **N** | **Sepsis median (IQR)** | **Mean rank (Sepsis)** | **N** | **No Sepsis median (IQR)** | **Mean rank (No Sepsis)** | **U** | **z** | **r** | **p-value** |
| --- | --- | --- | --- | --- | --- | --- | --- | --- | --- | --- |
| GFAP (pg/ml) D1 | 42 | 222.3 (137.8–274.7) | 30.19 | 19 | 206.1 (126.6–567.2) | 32.79 | 365 | -0.522 | -0.067 | 0.602 |
| *Brain Injury excluded | 38 | 235.1 (137.8–274.7) | 26.89 | 12 | 155.2 (122.6–220.5) | 21.08 | 281 | 1.193 | 0.169 | 0.233 |
| GFAP (pg/ml) D7 | 44 | 211.7 (124.8–427.9) | 28.66 | 16 | 291.3 (202.3–804.6) | 35.56 | 271 | -1.346 | -0.174 | 0.178 |
| *Brain Injury excluded | 30 | 194.4 (122.7–294.0) | 17.77 | 4 | 177.2 (116.8–245.9) | 15.5 | 68 | 0.401 | 0.069 | 0.688 |
| NFL (pg/ml) D1 | 40 | 221.5 (82.8–485.7) | 30.82 | 19 | 237.1 (111.4–419.9) | 28.26 | 413 | 0.527 | 0.069 | 0.598 |
| *Brain Injury excluded | 34 | 237.7 (88.2–480.5) | 24.85 | 12 | 196.2 (111.8–254.5) | 19.67 | 250 | 1.138 | 0.168 | 0.255 |
| NFL (pg/ml) D7 | 33 | 433.8 (281.8–783.9) | 23.24 | 11 | 361.1 (124.3–832.8) | 20.27 | 206 | 0.65 | 0.098 | 0.515 |
| *Brain Injury excluded | 26 | 504.7 (335.0–790.0) | 17.04 | 4 | 177.9 (123.2–296.3) | 5.5 | 92 | 2.41 | 0.44 | **0.016** |
| Tau (pg/ml) D1 | 48 | 8.3 (3.8–14.0) | 38.71 | 25 | 6.2 (3.7–11.1) | 33.72 | 682 | 0.947 | 0.111 | 0.343 |
| *Brain Injury excluded | 39 | 8.9 (3.9–14.1) | 27.21 | 12 | 6.1 (3.2–10.5) | 22.08 | 281 | 1.033 | 0.145 | 0.302 |
| Tau (pg/ml) D7 | 45 | 7.7 (4.1–12.0) | 31.91 | 16 | 5.7 (4.0–8.9) | 28.44 | 401 | 0.664 | 0.085 | 0.507 |
| *Brain Injury excluded | 30 | 7.5 (3.6–11.8) | 18.4 | 4 | 4.1 (2.1–5.7) | 10.75 | 87 | 1.416 | 0.243 | 0.157 |
| UCHL1 (pg/ml) D1 | 48 | 52.2 (29.7–106.1) | 38.94 | 25 | 46.8 (21.4–84.4) | 33.28 | 693 | 1.075 | 0.126 | 0.282 |
| *Brain Injury excluded | 39 | 49.5 (31.0–95.9) | 28.49 | 12 | 25.8 (8.7–62.5) | 17.92 | 331 | 2.143 | 0.3 | **0.032** |
| UCHL1 (pg/ml) D7 | 45 | 83.1 (46.9–123.0) | 32.07 | 16 | 64.2 (39.0–102.6) | 28 | 408 | 0.779 | 0.1 | 0.436 |
| *Brain Injury excluded | 30 | 78.4 (46.9–112.3) | 18.43 | 4 | 35.2 (22.9–66.2) | 10.5 | 88 | 1.47 | 0.252 | 0.142 |

*Values are median (IQR). Mean ranks, Mann–Whitney U, z (normal approximation with tie correction and continuity correction), and effect size r = z/sqrt(N) are reported. Two-sided p-values are derived from z.*

# Table 3.3.1 - Linear Regression Models Adjusted for Age and Age + Creatinine

| **Biomarker** | **N (age)** | **Beta (CI) age** | **p-value age** | **Fold Change age** | **Percent Change age** | **N (age+crea)** | **Beta (CI) age+crea** | **p-value age+crea** | **Fold Change age+crea** | **Percent Change age+crea** |
| --- | --- | --- | --- | --- | --- | --- | --- | --- | --- | --- |
| GFAP (pg/ml) D1 | 61 | -0.337 (-0.808, 0.134) | 0.157 | 0.714 | -28.634 | 61 | -0.376 (-0.878, 0.127) | 0.140 | 0.687 | -31.314 |
| *Brain Injury excluded | 50 | 0.136 (-0.323, 0.596) | 0.553 | 1.146 | 14.603 | 50 | 0.060 (-0.426, 0.545) | 0.806 | 1.061 | 6.144 |
| GFAP (pg/ml) D7 | 60 | -0.397 (-0.982, 0.187) | 0.179 | 0.672 | -32.798 | 60 | -0.213 (-0.829, 0.403) | 0.492 | 0.808 | -19.169 |
| *Brain Injury excluded | 34 | 0.046 (-0.786, 0.878) | 0.911 | 1.047 | 4.688 | 34 | 0.140 (-0.719, 1.000) | 0.741 | 1.151 | 15.082 |
| NFL (pg/ml) D1 | 59 | -0.026 (-0.544, 0.491) | 0.919 | 0.974 | -2.595 | 59 | -0.321 (-0.868, 0.227) | 0.246 | 0.726 | -27.426 |
| *Brain Injury excluded | 46 | 0.239 (-0.360, 0.838) | 0.426 | 1.270 | 26.978 | 46 | -0.041 (-0.645, 0.563) | 0.893 | 0.960 | -3.974 |
| NFL (pg/ml) D7 | 44 | 0.038 (-0.459, 0.534) | 0.879 | 1.038 | 3.826 | 44 | -0.142 (-0.665, 0.381) | 0.586 | 0.868 | -13.225 |
| *Brain Injury excluded | 30 | 0.811 (0.124, 1.498) | **0.022** | 2.250 | 125.018 | 30 | 0.613 (-0.048, 1.273) | 0.068 | 1.845 | 84.523 |
| Tau (pg/ml) D1 | 73 | 0.146 (-0.280, 0.572) | 0.497 | 1.157 | 15.692 | 73 | -0.179 (-0.608, 0.251) | 0.409 | 0.836 | -16.382 |
| *Brain Injury excluded | 51 | 0.271 (-0.269, 0.811) | 0.318 | 1.312 | 31.159 | 51 | -0.003 (-0.524, 0.517) | 0.990 | 0.997 | -0.341 |
| Tau (pg/ml) D7 | 61 | 0.159 (-0.327, 0.646) | 0.515 | 1.172 | 17.248 | 61 | 0.052 (-0.468, 0.573) | 0.841 | 1.054 | 5.378 |
| *Brain Injury excluded | 34 | 0.460 (-0.365, 1.284) | 0.264 | 1.583 | 58.332 | 34 | 0.268 (-0.541, 1.078) | 0.504 | 1.308 | 30.772 |
| UCHL1 (pg/ml) D1 | 73 | 0.250 (-0.281, 0.780) | 0.351 | 1.283 | 28.346 | 73 | -0.019 (-0.582, 0.545) | 0.947 | 0.981 | -1.861 |
| *Brain Injury excluded | 51 | 0.869 (0.203, 1.536) | **0.012** | 2.385 | 138.546 | 51 | 0.605 (-0.066, 1.275) | 0.076 | 1.830 | 83.039 |
| UCHL1 (pg/ml) D7 | 61 | 0.103 (-0.367, 0.574) | 0.662 | 1.109 | 10.865 | 61 | -0.054 (-0.549, 0.441) | 0.828 | 0.947 | -5.260 |
| *Brain Injury excluded | 34 | 0.560 (-0.223, 1.343) | 0.154 | 1.752 | 75.151 | 34 | 0.370 (-0.394, 1.134) | 0.331 | 1.447 | 44.743 |

*Biomarkers were log-transformed and analyzed using multivariable linear regression adjusted for age and age plus creatinine. β coefficients (95% CI) are shown; fold and percent changes were derived from exponentiated coefficients.*

# Table 3.4 - Biomarker Assessment (Mann–Whitney U Test) on Day 1 and Day 7 – mRS Subgroup

| **Biomarker** | **N (mRS ≥ 4)** | **mRS ≥ 4 median (IQR)** | **Mean rank (mRS ≥ 4)** | **N (mRS ≤ 3)** | **mRS ≤ 3 median (IQR)** | **Mean rank (mRS ≤ 3)** | **U** | **z** | **r** | **p-value** |
| --- | --- | --- | --- | --- | --- | --- | --- | --- | --- | --- |
| GFAP (pg/ml) D1 | 44 | 255.3 (162.5–407.4) | 35.82 | 17 | 136.5 (82.5–206.1) | 18.53 | 586 | 3.4 | 0.44 | **<0.001** |
| *Brain Injury excluded | 35 | 253.0 (150.2–317.2) | 29.83 | 15 | 136.5 (77.4–206.1) | 15.4 | 414 | 3.2 | 0.45 | **0.001** |
| GFAP (pg/ml) D7 | 50 | 256.9 (157.4–605.8) | 32.86 | 10 | 110.9 (89.9–227.7) | 18.7 | 368 | 2.33 | 0.3 | **0.020** |
| *Brain Injury excluded | 28 | 197.3 (129.1–299.1) | 18.79 | 6 | 110.9 (97.1–227.7) | 11.5 | 120 | 1.6 | 0.28 | 0.109 |
| NFL (pg/ml) D1 | 42 | 332.4 (132.9–510.6) | 35 | 17 | 85.0 (52.1–147.0) | 17.65 | 567 | 3.51 | 0.46 | **<0.001** |
| *Brain Injury excluded | 31 | 326.9 (186.0–480.5) | 27.97 | 15 | 111.4 (52.1–155.3) | 14.27 | 371 | 3.23 | 0.48 | **0.001** |
| NFL (pg/ml) D7 | 35 | 552.5 (332.5–922.1) | 25.06 | 9 | 178.7 (146.0–298.4) | 12.56 | 247 | 2.59 | 0.39 | **0.010** |
| *Brain Injury excluded | 24 | 504.7 (351.7–856.0) | 17.5 | 6 | 171.0 (146.0–231.5) | 7.5 | 120 | 2.46 | 0.45 | **0.014** |
| Tau (pg/ml) D1 | 53 | 8.9 (4.8–14.1) | 40.68 | 20 | 4.8 (2.8–8.7) | 27.25 | 725 | 2.41 | 0.28 | **0.016** |
| *Brain Injury excluded | 36 | 9.3 (4.9–15.1) | 28.78 | 15 | 5.3 (2.8–9.5) | 19.33 | 370 | 2.06 | 0.29 | **0.040** |
| Tau (pg/ml) D7 | 51 | 7.7 (4.3–14.4) | 33.96 | 10 | 3.3 (2.1–4.7) | 15.9 | 406 | 2.93 | 0.38 | **0.003** |
| *Brain Injury excluded | 28 | 7.5 (4.3–13.1) | 19.43 | 6 | 2.7 (1.6–4.3) | 8.5 | 138 | 2.42 | 0.41 | **0.016** |
| UCHL1 (pg/ml) D1 | 53 | 70.9 (41.4–111.3) | 43.32 | 20 | 23.9 (6.4–39.1) | 20.25 | 865 | 4.14 | 0.48 | **<0.001** |
| *Brain Injury excluded | 36 | 55.3 (35.7–96.8) | 31 | 15 | 22.3 (6.5–36.3) | 14 | 450 | 3.71 | 0.52 | **<0.001** |
| UCHL1 (pg/ml) D7 | 51 | 84.1 (61.4–151.1) | 33.94 | 10 | 22.9 (18.7–53.2) | 16 | 405 | 2.91 | 0.37 | **0.004** |
| *Brain Injury excluded | 28 | 82.8 (58.7–107.6) | 19.39 | 6 | 22.9 (21.4–40.3) | 8.67 | 137 | 2.37 | 0.41 | **0.018** |

*Values are median (IQR). Mean ranks, Mann–Whitney U, z (normal approximation with tie correction and continuity correction), and effect size r = z/sqrt(N) are reported. Two-sided p-values are derived from z.*

# Table 3.4.1 - Linear Regression Models Adjusted for Age and Age + Creatinine

| **Biomarker** | **N (age)** | **Beta (CI) age** | **p-value age** | **Fold Change age** | **Percent Change age** | **N (age+crea)** | **Beta (CI) age+crea** | **p-value age+crea** | **Fold Change age+crea** | **Percent Change age+crea** |
| --- | --- | --- | --- | --- | --- | --- | --- | --- | --- | --- |
| GFAP (pg/ml) D1 | 61 | 0.742 (0.274, 1.211) | **0.002** | 2.101 | 110.101 | 61 | 0.748 (0.274, 1.221) | **0.003** | 2.112 | 111.189 |
| *Brain Injury excluded | 50 | 0.555 (0.137, 0.972) | **0.010** | 1.741 | 74.109 | 50 | 0.533 (0.112, 0.955) | **0.014** | 1.704 | 70.446 |
| GFAP (pg/ml) D7 | 60 | 0.678 (-0.027, 1.382) | 0.059 | 1.969 | 96.905 | 60 | 0.730 (0.048, 1.412) | **0.036** | 2.075 | 107.521 |
| *Brain Injury excluded | 34 | 0.244 (-0.510, 0.999) | 0.514 | 1.276 | 27.650 | 34 | 0.259 (-0.499, 1.017) | 0.491 | 1.295 | 29.533 |
| NFL (pg/ml) D1 | 59 | 0.845 (0.358, 1.332) | **0.001** | 2.328 | 132.847 | 59 | 0.810 (0.338, 1.282) | **0.001** | 2.248 | 124.814 |
| *Brain Injury excluded | 46 | 0.787 (0.249, 1.326) | **0.005** | 2.197 | 119.714 | 46 | 0.695 (0.182, 1.208) | **0.009** | 2.004 | 100.350 |
| NFL (pg/ml) D7 | 44 | 0.544 (0.020, 1.068) | **0.042** | 1.723 | 72.289 | 44 | 0.523 (0.010, 1.036) | **0.046** | 1.687 | 68.737 |
| *Brain Injury excluded | 30 | 0.717 (0.080, 1.355) | **0.029** | 2.049 | 104.909 | 30 | 0.661 (0.093, 1.229) | **0.024** | 1.936 | 93.615 |
| Tau (pg/ml) D1 | 73 | 0.450 (0.001, 0.899) | **0.049** | 1.569 | 56.883 | 73 | 0.372 (-0.047, 0.790) | 0.081 | 1.450 | 45.007 |
| *Brain Injury excluded | 51 | 0.422 (-0.095, 0.938) | 0.107 | 1.525 | 52.467 | 51 | 0.333 (-0.139, 0.805) | 0.163 | 1.395 | 39.511 |
| Tau (pg/ml) D7 | 61 | 0.791 (0.229, 1.352) | **0.007** | 2.205 | 120.513 | 61 | 0.768 (0.207, 1.329) | **0.008** | 2.155 | 115.460 |
| *Brain Injury excluded | 34 | 0.676 (-0.051, 1.403) | 0.067 | 1.966 | 96.622 | 34 | 0.644 (-0.040, 1.327) | 0.064 | 1.903 | 90.324 |
| UCHL1 (pg/ml) D1 | 73 | 1.195 (0.694, 1.697) | **0.000** | 3.305 | 230.506 | 73 | 1.134 (0.645, 1.622) | **0.000** | 3.107 | 210.705 |
| *Brain Injury excluded | 51 | 1.182 (0.579, 1.784) | **0.000** | 3.259 | 225.944 | 51 | 1.084 (0.526, 1.642) | **0.000** | 2.957 | 195.660 |
| UCHL1 (pg/ml) D7 | 61 | 0.888 (0.360, 1.416) | **0.001** | 2.430 | 143.049 | 61 | 0.857 (0.336, 1.377) | **0.002** | 2.355 | 135.525 |
| *Brain Injury excluded | 34 | 0.851 (0.181, 1.521) | **0.015** | 2.341 | 134.127 | 34 | 0.818 (0.200, 1.435) | **0.011** | 2.265 | 126.508 |

*Biomarkers were log-transformed and analyzed using linear regression with Outcome_mRS (0 = good, 1 = poor outcome) as predictor, adjusted for age and age plus creatinine. β coefficients (95% CI) are shown; fold and percent changes were derived from exponentiated coefficients.*

# Table 4.1 - Discriminatory Performance (AUC and ΔAUC) of Model 1 (Age+SOFA)

| **Risk Factors** | **Unfavorable Outcome (mRS ≥4) AUC** | **p-Value** | **Mortality on ICU AUC** | **p-Value** | **Mortality on Day 90 AUC** | **p-Value** |
| --- | --- | --- | --- | --- | --- | --- |
| Reference (Age+SOFA) | 0.841 (0.742–0.930) | **<.001** | 0.814 (0.707–0.905) | **0.001** | 0.756 (0.639–0.862) | **0.002** |
| + GFAP | 0.880 (0.779–0.965) | **<.001** | 0.800 (0.678–0.904) | **0.011** | 0.775 (0.654–0.881) | **0.007** |
| + Delta AUC | 0.029 (-0.006–0.096) | 0.131 | -0.000 (-0.024–0.088) | 0.690 | -0.004 (-0.021–0.091) | 0.777 |
| + Nfl | 0.857 (0.739–0.952) | **<.001** | 0.773 (0.647–0.880) | **0.047** | 0.713 (0.564–0.849) | 0.192 |
| + Delta AUC | 0.027 (-0.011–0.136) | 0.323 | -0.000 (-0.022–0.189) | 0.485 | 0.012 (-0.030–0.169) | 0.582 |
| + Tau | 0.864 (0.766–0.948) | **<.001** | 0.821 (0.706–0.914) | **0.006** | 0.765 (0.634–0.881) | **0.013** |
| + Delta AUC | 0.010 (-0.010–0.045) | 0.604 | 0.014 (-0.015–0.053) | 0.703 | 0.021 (-0.015–0.069) | 0.515 |
| + UCHL-1 | 0.881 (0.785–0.957) | **<.001** | 0.850 (0.749–0.937) | **0.003** | 0.783 (0.655–0.896) | **0.012** |
| + Delta AUC | 0.027 (-0.006–0.123) | 0.147 | 0.042 (-0.008–0.107) | 0.232 | 0.039 (-0.009–0.100) | 0.290 |
| + (GFAP+UCHL-1) | 0.910 (0.826–0.973) | **<.001** | 0.831 (0.720–0.924) | **0.014** | 0.809 (0.688–0.906) | **0.008** |
| + Delta AUC | 0.060 (0.009–0.165) | **0.016** | 0.031 (-0.014–0.149) | 0.211 | 0.029 (-0.014–0.141) | 0.199 |
| + All Biomarkers | 0.897 (0.801–0.969) | **<.001** | 0.846 (0.742–0.939) | 0.138 | 0.810 (0.682–0.920) | 0.085 |
| + Delta AUC | 0.057 (0.013–0.191) | **0.015** | 0.056 (-0.009–0.228) | 0.100 | 0.039 (-0.012–0.214) | 0.113 |

*AUCs are reported with 95% confidence intervals obtained by bootstrap resampling (2000 iterations). Overall model p-values were derived from likelihood ratio tests comparing each model with an intercept-only model. Differences in AUC (ΔAUC) were calculated on paired complete cases. Confidence intervals for ΔAUC were estimated using paired bootstrap resampling (2000 iterations), and two-sided p-values were derived from the empirical bootstrap distribution.*

# Table 4.2 - Discriminatory Performance (AUC and ΔAUC) of Model 2 (Age+GCS)

| **Risk Factors** | **Unfavorable Outcome (mRS ≥4) AUC** | **p-Value** | **Mortality on ICU AUC** | **p-Value** | **Mortality on Day 90 AUC** | **p-Value** |
| --- | --- | --- | --- | --- | --- | --- |
| Reference (Age+GCS) | 0.869 (0.770–0.944) | **<.001** | 0.751 (0.609–0.873) | **0.003** | 0.705 (0.579–0.823) | **0.005** |
| + GFAP | 0.926 (0.848–0.983) | **<.001** | 0.811 (0.689–0.920) | **0.006** | 0.783 (0.664–0.890) | **0.005** |
| + Delta AUC | 0.014 (-0.007–0.060) | 0.302 | 0.013 (-0.016–0.074) | 0.604 | 0.018 (-0.015–0.084) | 0.501 |
| + Nfl | 0.912 (0.837–0.972) | **<.001** | 0.816 (0.687–0.927) | **0.024** | 0.709 (0.557–0.842) | 0.081 |
| + Delta AUC | 0.008 (-0.012–0.069) | 0.590 | 0.023 (-0.022–0.155) | 0.562 | -0.006 (-0.030–0.131) | 0.721 |
| + Tau | 0.900 (0.812–0.965) | <.001 | 0.820 (0.694–0.923) | **0.001** | 0.777 (0.660–0.875) | **0.003** |
| + Delta AUC | 0.002 (-0.010–0.024) | 0.829 | 0.007 (-0.014–0.055) | 0.648 | 0.018 (-0.012–0.064) | 0.494 |
| + UCHL-1 | 0.903 (0.809–0.972) | **<.001** | 0.839 (0.723–0.928) | **<.001** | 0.780 (0.657–0.884) | **0.002** |
| + Delta AUC | 0.005 (-0.012–0.067) | 0.492 | 0.026 (-0.008–0.087) | 0.245 | 0.022 (-0.012–0.087) | 0.297 |
| + (GFAP+UCHL-1) | 0.936 (0.868–0.983) | **<.001** | 0.838 (0.718–0.938) | **0.006** | 0.812 (0.693–0.909) | **0.005** |
| + Delta AUC | 0.023 (0.000–0.101) | 0.051 | 0.040 (-0.012–0.124) | 0.181 | 0.047 (-0.008–0.123) | 0.123 |
| + All Biomarkers | 0.926 (0.850–0.980) | **<.001** | 0.828 (0.697–0.934) | 0.085 | 0.812 (0.690–0.909) | 0.089 |
| + Delta AUC | 0.024 (0.003–0.142) | **0.038** | 0.032 (-0.009–0.185) | 0.130 | 0.081 (-0.002–0.234) | 0.063 |

*AUCs are reported with 95% bootstrap confidence intervals (2000 resamples). Overall model p-values were derived from likelihood ratio tests versus intercept-only models. Differences in AUC (ΔAUC) were calculated on paired complete cases, with 95% confidence intervals obtained by paired bootstrap resampling.*

# Table 4.3 - Discriminatory Performance (AUC and ΔAUC) of Model 3 (APACHE II)

| **Risk Factors** | **Unfavorable Outcome (mRS ≥4) AUC** | **p-Value** | **Mortality on ICU AUC** | **p-Value** | **Mortality on Day 90 AUC** | **p-Value** |
| --- | --- | --- | --- | --- | --- | --- |
| Reference (Apache) | 0.760 (0.652–0.855) | **<.001** | 0.857 (0.732–0.949) | **<.001** | 0.821 (0.715–0.911) | **<.001** |
| + GFAP | 0.853 (0.739–0.941) | **<.001** | 0.835 (0.700–0.942) | **<.001** | 0.839 (0.723–0.935) | **<.001** |
| + Delta AUC | 0.077 (-0.001–0.200) | 0.052 | 0.001 (-0.013–0.047) | 0.688 | 0.003 (-0.011–0.053) | 0.483 |
| + Nfl | 0.803 (0.662–0.917) | **<.001** | 0.853 (0.691–0.971) | **<.001** | 0.824 (0.691–0.933) | **<.001** |
| + Delta AUC | 0.058 (-0.006–0.217) | 0.102 | -0.003 (-0.022–0.082) | 0.699 | -0.003 (-0.022–0.064) | 0.706 |
| + Tau | 0.782 (0.673–0.879) | **<.001** | 0.850 (0.714–0.958) | **<.001** | 0.843 (0.721–0.935) | **<.001** |
| + Delta AUC | 0.006 (-0.016–0.104) | 0.395 | -0.012 (-0.026–0.059) | 0.979 | 0.005 (-0.018–0.054) | 0.675 |
| + UCHL-1 | 0.840 (0.731–0.930) | **<.001** | 0.885 (0.772–0.967) | **<.001** | 0.852 (0.734–0.941) | **<.001** |
| + Delta AUC | 0.063 (0.001–0.168) | 0.047 | 0.023 (-0.006–0.077) | 0.201 | 0.013 (-0.009–0.073) | 0.326 |
| + (GFAP+UCHL-1) | 0.900 (0.811–0.965) | **<.001** | 0.855 (0.722–0.949) | **<.001** | 0.865 (0.752–0.952) | **<.001** |
| + Delta AUC | 0.124 (0.046–0.261) | **<.001** | 0.021 (-0.010–0.093) | 0.200 | 0.029 (-0.005–0.106) | 0.116 |
| + All Biomarkers | 0.891 (0.786–0.969) | **<.001** | 0.874 (0.720–0.977) | **0.007** | 0.887 (0.764–0.970) | **0.002** |
| + Delta AUC | 0.134 (0.056–0.308) | **0.003** | 0.023 (-0.002–0.162) | 0.068 | 0.039 (0.004–0.159) | **0.035** |

*AUCs are reported with 95% bootstrap confidence intervals (2000 resamples). Overall model p-values were derived from likelihood ratio tests versus intercept-only models. Differences in AUC (ΔAUC) were calculated on paired complete cases, with 95% confidence intervals obtained by paired bootstrap resampling.*

# Table 5.1 - Univariable Logistic Regression Models

| **Biomarker/Variable** | **Univariable Regression OR (CI)** | **p- Value** |
| --- | --- | --- |
| Age | 1.03 (1.00-1.07) | 0.075 |
| Sex (ref:male) | 0.51 (0.19-1.41) | 0.191 |
| Brain Injury | 2.16 (0.78-6.67) | 0.153 |
| Log Creatinine D1 | 2.20 (0.95-5.60) | 0.078 |
| Log GFAP D1 | 6.60 (2.27-26.51) | **0.002** |
| Log NFL D1 | 3.78 (1.82-9.26) | **0.001** |
| Log Tau D1 | 2.37 (1.22-5.04) | **0.016** |
| Log UCHL-1 D1 | 4.59 (2.26-11.67) | **<0.001** |

*Represents the OR of mRS ≥4 vs. mRS ≤3*

# Table 5.2 - Multivariable Logistic Regression Models

| Biomarker/Variable | Model 1 OR (CI) | p- Value | Model 2 OR (CI) | p- Value | Model 3 OR (CI) | p- Value | Model 4 OR (CI) | p- Value |
| --- | --- | --- | --- | --- | --- | --- | --- | --- |
| Age | 1.04 (0.98-1.13) | 0.234 | 1.04 (0.98-1.12) | 0.207 | 1.05 (1.00-1.11) | 0.086 | 1.02 (0.96-1.09) | 0.475 |
| Brain Injury | 2.03 (0.25-22.99) | 0.524 | 8.77 (1.14-108.68) | 0.056 | 2.99 (0.76-14.11) | 0.136 | 1.42 (0.24-9.07) | 0.697 |
| Log Creatinine D1 | 1.41 (0.38-5.56) | 0.608 | 0.88 (0.19-3.81) | 0.863 | 1.51 (0.46-5.11) | 0.496 | 0.97 (0.22-4.05) | 0.967 |
| Log GFAP D1 | **5.11 (1.57-22.33)** | **0.015** |  |  |  |  |  |  |
| Log NFL D1 |  |  | **3.87 (1.57-12.05)** | **0.008** |  |  |  |  |
| Log Tau D1 |  |  |  |  | 1.73 (0.82-3.95) | 0.169 |  |  |
| Log UCHL-1 D1 |  |  |  |  |  |  | **4.17 (1.90-11.35)** | **0.001** |

*Represents the OR of mRS ≥4 vs. mRS ≤3*
